# Ecology of reproduction of *Anopheles arabiensis* in an urban area of Bobo-Dioulasso, Burkina Faso (West Africa): Monthly swarming and mating frequency and their relation to environmental factors

**DOI:** 10.1371/journal.pone.0205966

**Published:** 2018-11-07

**Authors:** Nanwintoum Séverin Bimbilé Somda, Bèwadéyir Serge Poda, Péguédwindé Simon Sawadogo, Olivier Gnankiné, Hamidou Maiga, Florence Fournet, Rosemary Susan Lees, Jeremy Bouyer, Jérémie Gilles, Antoine Sanon, Abdoulaye Diabaté, Kounbobr Roch Dabiré

**Affiliations:** 1 Institut de Recherche en Sciences de la Santé/Direction Régionale de l’Ouest (IRSS/DRO), Bobo-Dioulasso, Burkina Faso; 2 Laboratoire d’Entomologie Fondamentale et Appliquée, Université Ouaga 1 Joseph Ki-Zerbo, Ouagadougou, Burkina Faso; 3 Unité maladies infectieuses et vecteurs: écologie, génétique, évolution et contrôle, Institut de Recherche pour le Développement (IRD), Montpellier, France; 4 Liverpool Insect Testing Establishment (LITE), Vector Biology Department, Liverpool School of Tropical Medicine, Pembroke Place, Liverpool, L3 5QA, United Kingdom; 5 Insect Pest Control Laboratory, International Atomic Energy Agency, Joint FAO/IAEA, Vienna, Austria; Universita degli Studi di Camerino, ITALY

## Abstract

Swarming is a key part of the natural system of reproduction of anopheline mosquito populations, and a better understanding of swarming and mating systems in a targeted species in its natural habitat would contribute to better design control strategies with a greater chance of success. Our study investigated the monthly occurrence of swarming and the mating frequency (within swarms) of *Anopheles arabiensis* in Dioulassoba, Burkina Faso and their relationship with local environmental factors. Mosquitoes collected from swarms were described in terms of body size, recent sugar meal intake, and female repletion, insemination, and *Plasmodium falciparum* infection status. Swarms of *An*. *arabiensis* were found in each month of the year. Both start and end times of swarming varied significantly between months, correlating with the time of sunset. Swarming mostly started after or coincided with sunset from late July to early October but occurred before sunset from late October to early July. Swarming duration, the number of mosquitoes and mating pairs per swarm, and time to first mating were significantly different between months in an inverse relationship with the monthly rainfall. The number of mating pairs was strongly and positively correlated with swarm size. Almost all the females caught *in copula* were inseminated but a very few were blood fed; no *P*. *falciparum* infection was observed. Males caught *in copula* and *in solo* were similar in body size and in the proportion which had taken a recent sugar meal. Our investigations showed that *An*. *arabiensis* reproductive activities are most frequent during the dry season, suggesting either the species’ preference for dry climatic conditions or a lack of available breeding sites during the rainy season due to the seasonal flooding in this area. Targeting interventions to kill mosquitoes in swarms or to achieve an over-flooding ratio of sterile males during the rainy season would increase their efficiency in reducing the population density of this vector.

## Introduction

Malaria remains one of the most challenging vector-borne diseases to control even though its incidence was estimated to have fallen by around 41% globally between 2000 and 2015 [1]. While efforts are devoted to finding an effective vaccine, the prevention of this disease mainly relies on management of its vectors, through reducing human-vector contact and vector survival [2]. Research on vector control has historically focused on the female mosquito, which is responsible for parasite transmission in humans. However, novel strategies of control are based on mating biology [3–6], so male biology must also be considered and a better understanding of the ecology of mosquito reproduction is crucial.

The mating swarm is a key feature of male biology in many mosquito species, an aggregate of males into which individual females enter for mating [7], and as such is a key part of the natural system of reproduction. Understanding the ecology of swarming and mating gives us a greater understanding of mosquito species and deepening our knowledge of the mechanisms and environmental variables controlling the mating behavior can help to refine existing intervention tools and help in developing new ones. Several studies have investigated the swarming systems of different species belonging to the genera *Anopheles* [8–13], *Aedes* [14] and *Culex* [15]. Such investigations [13, 16] have contributed to the reclassification of two incipient species, *An*. *gambiae* M and S forms, into distinct species, *An*. *coluzzii* and *An*. *gambiae*, respectively [17, 18]. Based more specifically on the male swarming system, Diabaté *et al*. [19] described two potential interventions to control malaria: the development of a sound or chemical trap that makes use of putative sensory cues used by *An*. *gambiae* in swarm formation, and a lure-and-kill strategy exploiting visual cues involved in swarming. Some promising alternative mosquito control techniques rely on understanding and exploiting mating behaviour, including the sterile insect technique, of which *An*. *arabiensis* is one species currently being targeted [20–23]. Knowing that the ecology of mosquito reproduction is impacted by environmental factors including climate-based and man-made factors [24], the development and/or implementation of control tools relying on male biology requires a deep understanding of the swarm system of the target species in the target area.

*An*. *arabiensis* is one of the major vectors in the African WHO (World Health Organization) malaria region, with a wide distribution including arid and urban areas [18]. In Burkina Faso, this species has recently been found to be the most significant malaria vector in a Sudanese zone, preciously in an urban area of Bobo-Dioulasso [25] where it comprised only a small percentage of the total *An*. *gambiae s*.*l*. population throughout the year. [26]. Given its surprising adaptation to this area, a study was recently carried out into the occurrence of *An*. *arabiensis* swarms for the first time in this country [27]. This study targeted short periods in September, October, March and April, investigating basic parameters such as visual markers, the number of swarms, their height and swarming start and end times [27]. Thus, little is now known about the swarming and mating dynamics over the course of a year and in relation to the seasons, and it is clear that this species favours arid climatic conditions. To build on this knowledge, the present study investigated the monthly occurrence of swarms, the dynamics of mating within swarms, and their relationship with local environmental factors, including time of sunset, total hours of sunshine, rainfall, rain frequency and minimum, maximum and average temperatures. The males caught *in copula* were compared to their counterparts caught *in solo* in terms of body size and presence or absence of a recent sugar meal. Additionally, females collected *in copula* were checked for repletion, insemination and *Plasmodium falciparum* infection status.

## Materials and methods

### The study site

The study was carried out in Dioulassoba (11°10'42 "N; 4°17'35" W), a central district of Bobo-Dioulasso, Burkina Faso, located in the south-west of the country in a Sudanese savannah region. The study area measures 550 m by 160 m, located along the Houet river, and is used to grow vegetable crops which are sprayed systematically with pesticides by residents, who also breed domestic animals such as pigs, poultry, sheep, goats, donkeys, oxen and dogs. The Houet river is a year-round source of water flowing through Dioulassoba, polluted by household garbage and animal and human sewage.

### *Anopheles arabiensis* swarming and mating parameters over the course of a year in Dioulassoba

The study was conducted over the course of a year, from July 2013 to June 2014, with observations being made for *An*. *arabiensis* swarms every month. A sample of swarms was selected to represent the diversity of the markers (bare soil, roof, sand, wood bundles, garbage and patches of leaking water from toilets) and achieve an even coverage of the study area. As we could not observe many swarms above each type of marker and because some swarms did not occur on every observation day and in every month, we considered at least one swarming site per day above each marker type when possible to consider all types of marker. Seven to ten swarming sites were explored per day of observation and fifteen to twenty swarming sites per month, mostly targeting potentially permanent swarms. Observations were conducted on at least 3 evenings per week. For each swarm, the following parameters were recorded: the times when swarms formed and dispersed, the duration of swarming, the number of mosquitoes, the number of mating pairs, and the time between swarm formation and the first observed mating. The time when swarming started (swarm formation) was defined as the time that the first male appeared at the swarm site, and the end of swarming (dispersal) defined as the time at which no mosquito was seen involved in the regular movement characteristic of swarming. In general, the swarming ended before it became completely dark but in some cases, we used camera´s flash around the expected swarming end time to determine that time more accurately. In addition, the personnel conducting the swarm observations had experience, and to minimize the bias, at least two people were allocated to each swarming site. The duration of swarming was the period of times between these events. To determine the number of mosquitoes per swarm throughout the swarm period, photos were taken from swarm formation until swarm dispersal. Images with the highest apparent mosquito density were assumed to represent the maximum number of mosquitoes in the swarm and were selected for counting. The number of mating-pairs per swarm was determined by direct counting in real time by an observer using a manual counter (Hand tally counter, UNIWISE, Zhejiang, China). The time of the first observed mating pair was recorded and the time between the swarm formation and the first mating subsequently calculated.

### Relationship between *An*. *arabiensis* swarming and mating dynamics and sunset time, temperature, rainfall, rain frequency and time of sunshine in Dioulassoba

The local sunset data were collected from a website (http://www.sunrise-and-sunset.com/en). The data on local monthly temperature, total rainfall, days of rain and time of sunshine were recorded by the weather station of Bobo-Dioulasso Airport. The monthly rain frequency was calculated from the number of rainy days recorded during the study as a proportion of the total number of days of the month. Monthly sunshine was defined as the number of hours of sunshine in the month. To more accurately determine their impact on the swarming dynamics, temperature and relative humidity were also recorded at the start and the end of the swarming period each time observations were made between August 2013 and April 2014, using a data logger (Model: ETHG912, OREGON Scientific, Tualatin, Oregon, U.S.A.).

### Characterization of mosquitoes collected from swarms: Female repletion, insemination, *Plasmodium falciparum* infection status, body size and presence of a recent sugar meal

Mating-pairs sampled from swarms, collected using a sweep-net, were immediately placed on ice and sent to the laboratory. Repletion status of females was recorded. Spermathecae were then dissected under a binocular microscope and insemination status determined under a microscope at ×40 objective, and insemination rate determined from the number of inseminated females as a proportion of the total number of successfully dissected females. A sample of females caught *in copula* was analyzed using a circumsporozoite protein enzyme-linked immunosorbent assay (CSP-ELISA) test [28] to determine the *P*. *falciparum* infection rate.

Mosquito body size was estimated through wing length measurement. The left wings of males collected *in solo* and males and females collected *in copula* were dissected and mounted between glass slides. Photos of the wings were captured using a Leica microscope equipped with LAZ 2.1.0. Camera software, and stored on a computer. The lengths from the alula notch to the wing tip [29] were measured using Image J 1. 42q. software.

The presence or absence of recently ingested sugar in the abdomens of males collected *in solo* and males and females collected *in copula* from the same swarms was determined using the Cold-Anthrone Test [30], which reveals the presence of the disaccharide sucrose (or its components glucose and fructose) obtained from nectar and fruit juices and stored in the crop [30]. The proportion of freshly sugar-fed mosquitoes was estimated from the number of positive individuals as a proportion of the total number successfully tested in each group.

### Molecular analysis

A sample of mosquitoes from all the swarms investigated (30 males per swarm) was identified by species using the molecular identification protocol of Santolomazza *et al*. [31].

### Statistical analysis

Swarming and mating parameters and climatic conditions were compared between months, using generalized linear model (GLNM) analysis and their correlations tested using Pearson’s correlation test (2-tailed) with significance at the 0.05 level, in IBM SPSS statistics 22 software (IBM Corp. Released 2013. IBM SPSS Statistics for Windows, Version 22.0. Armonk, NY: IBM Corp.). The Gamma distribution with log link function was considered for the dependent variables sunset time, start and end times of swarming, duration of swarming, time to the first observed mating from the swarm formation and mosquito wing length; for the dependent variables number of mosquitoes and number of mating pairs per swarm, the negative binomial distribution with log link function was used. The model including intercept and month as predictor and, analyze type III with 95% Wald confidence interval were considered. Pairwise comparisons with sequential Bonferroni correction were performed; the mean difference was significant at the 0.05 level. The proportion of mosquitoes which had recently taken a sugar meal was compared between groups using the Chi-squared test. Graphs were made using IBM SPSS statistics 22 software.

### Ethics statement

The study did not involve vertebrates. For any locations/activities the authors state clearly that permission has been obtained from local authorities (the traditional authorities of Dioulassoba) before the start of the study. Field studies did not involve endangered or protected species.

## Results

### *Anopheles arabiensis* swarming and mating parameters over the course of a year in Dioulassoba

**Fig 1:**
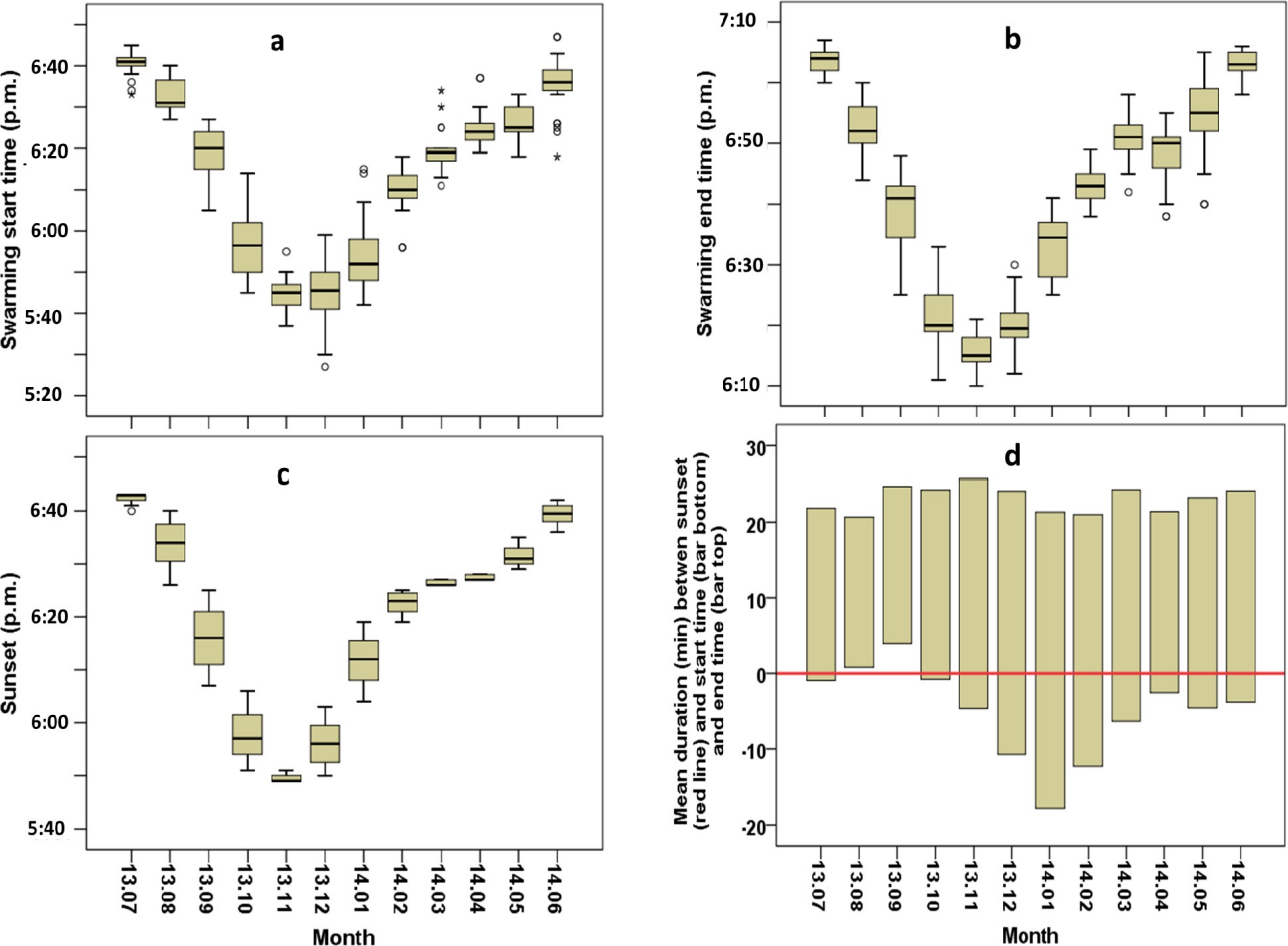
Monthly *Anopheles arabiensis* swarming times in Dioulassoba. (a) Swarming start time. (b) Swarming end time. (c) Sunset time. (d) Mean duration between sunset and swarming start and end times.

Molecular analysis has shown that all the *Anopheles* swarms found in the study period exclusively comprised *An*. *arabiensis*. Swarms of *An*. *arabiensis* were found each month of the year, starting between 05:27 and 06:45 p.m. and ending between 06:10 and 07:05 p.m. Both start and end time varied significantly between months (respectively GLNM, df = 11, Wald χ^2^ = 8797.57, *P*<0.001 and GLNM, df = 11, Wald χ^2^ = 13830.38, *P*<0.001, Tables 1 and 2), becoming earlier from July 2013 to December 2013 and later from December 2013 to June 2014 (Fig 1A and 1B), following the variation in sunset time (Fig 1C) which also differed between months (GLNM, df = 11, Wald χ^2^ = 94596.97, *P*<0.001, Table 3). The relationship between swarming and sunset timing depended on the period; from late July to early October swarming was mostly found to start after or coinciding with the sunset but swarming occurred before sunset from late October to early July (Fig 1D).

**Table 1:** Estimated marginal means of swarming start time following the month.

Month	N	Mean	Standard error	95% Wald Confidence Interval	
				Lower	Upper
13.07	33	1120.82 a	0.46	1119.92	1121.72
14.06	35	1115.26 b	1.12	1113.07	1117.45
13.08	32	1112.62 b	0.68	1111.30	1113.96
14.05	37	1106.57 c	0.58	1105.42	1107.71
14.04	41	1104.63 c	0.61	1103.43	1105.84
14.03	33	1099.94 d	0.94	1098.11	1101.77
13.09	32	1098.69 d	1.11	1096.52	1100.86
14.02	35	1089.83 e	0.82	1088.23	1091.43
13.10	68	1076.41 f	0.87	1074.70	1078.12
14.01	38	1073.95 f	1.32	1071.37	1076.54
13.11	34	1065.24 g	0.62	1064.02	1066.46
13.12	62	1065.24 g	0.83	1063.63	1066.86

Different letters indicate statistically significant differences between groups (Sequential Bonferroni pairwise comparisons; the mean difference is significant at the 0.05 level).

**Table 2:** Estimated marginal means of swarming end time following the month.

Month		Mean	Standard error	95% Wald Confidence Interval	
	N			Lower	Upper
13.07	33	1143.61 a	0.34	1142.94	1144.27
14.06	35	1143.20 a	0.41	1142.40	1144.00
14.05	37	1134.35 b	0.92	1132.55	1136.16
13.08	32	1132.47 bc	0.75	1131.00	1133.93
14.03	33	1130.55 cd	0.53	1129.50	1131.59
14.04	41	1128.59 d	0.67	1127.40	1129.77
14.02	35	1123.14 e	0.47	1122.22	1124.07
13.09	32	1119.44 f	0.96	1117.57	1121.31
14.01	38	1113.05 g	0.74	1111.60	1114.51
13.10	68	1101.43 h	0.57	1100.31	1102.55
13.12	62	1100.05 h	0.474	1099.12	1100.98
13.11	34	1095.53 i	0.476	1094.60	1096.46

Different letters indicate statistically significant differences between groups (Sequential Bonferroni pairwise comparisons; the mean difference is significant at the 0.05 level).

**Table 3:** Estimated marginal means of sunset time following the month.

Month	N	Mean	Standard error	95% Wald Confidence Interval	
				Lower	Upper
13.07	33	1121.76 a	0.22	1121.33	1122.19
14.06	35	1119.09 b	0.29	1118.52	1119.65
13.08	32	1111.81 c	0.79	1110.27	1113.36
14.05	37	1111.14 c	0.31	1110.54	1111.74
14.04	41	1107.20 d	0.06	1107.07	1107.32
14.03	33	1106.27 e	0.09	1106.10	1106.45
14.02	35	1102.14 f	0.38	1101.40	1102.89
13.09	32	1094.75 g	0.82	1093.14	1096.37
14.01	38	1091.74 h	0.62	1090.52	1092.95
13.10	68	1077.19 i	0.45	1076.30	1078.08
13.12	62	1075.97 i	0.45	1075.08	1076.86
13.11	34	1069.88 j	0.12	1069.64	1070.12

Different letters indicate statistically significant differences between groups (Sequential Bonferroni pairwise comparisons; the mean difference is significant at the 0.05 level)

**Fig 2:**
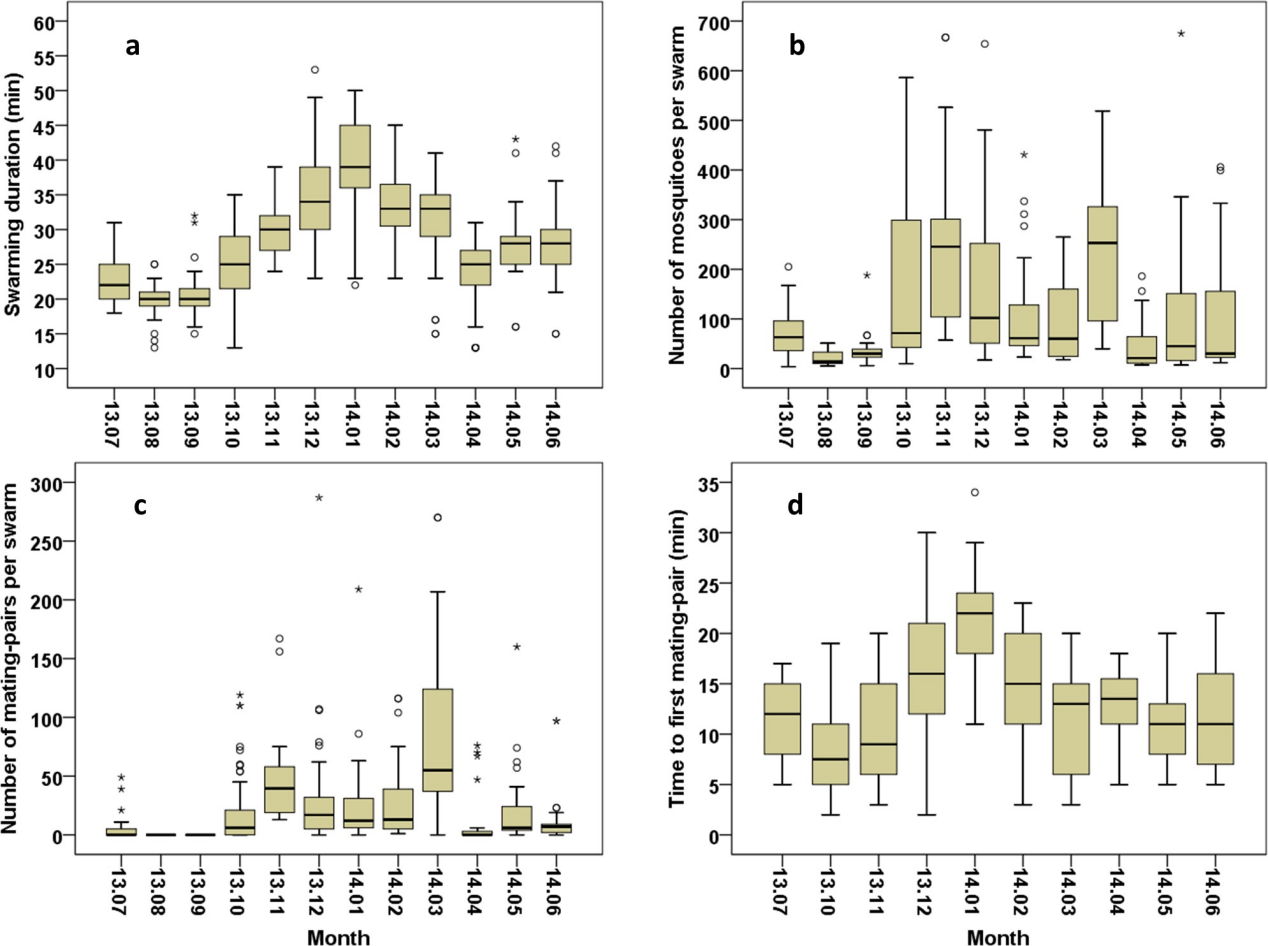
Monthly *Anopheles arabiensis* swarming and mating parameters in Dioulassoba. (a)Swarm duration. (b) Swarm size (c) Number of mating-pairs per swarm. (d) Time from swarm formation to first mating.

The swarm durations (Fig 2A) were comprised between 13 and 53 minutes and were significantly different between the months, with the shortest swarms observed during August, September and April and the longest in December and January (GLNM, df = 11, Wald χ^2^ = 699.924, *P*<0.001, Table 4). Overall, the mean duration was 28.31±0.35 minutes (N = 480).

**Table 4:** Estimated marginal means of swarming duration following the month.

Month	N	Mean	Standard error	95% Wald Confidence Interval	
				Lower	Upper
14.01	38	39.11 a	1.10	37.01	41.32
13.12	62	34.81 b	0.80	33.27	36.41
14.02	35	33.31 bc	0.92	31.57	35.16
14.03	33	30.61 cd	1.14	28.45	32.93
13.11	34	30.29 cd	0.80	28.77	31.90
14.06	35	27.94 de	1.01	26.03	30.00
14.05	37	27.78 de	0.82	26.22	29.44
13.10	68	25.01 ef	0.64	23.79	26.30
14.04	41	23.95 f	0.77	22.48	25.51
13.07	33	22.79 fg	0.57	21.69	23.94
13.09	32	20.75 gh	0.65	19.52	22.05
13.08	32	19.84 h	0.46	18.96	20.77

Different letters indicate statistically significant differences between groups (Sequential Bonferroni pairwise comparisons; the mean difference is significant at the 0.05 level).

The swarm size, defined as the number of mosquitoes per swarm (Fig 2B), depended on the month (GLNM, df = 11, Wald χ2 = 401.977, *P*<0.001, Table 5) and ranged between 4 and 700 mosquitoes. The lowest swarm sizes mostly recorded during the period of August to September and in April and the highest sizes mainly observed in November and March. The mean swarm size was 118.55±0.65 (N = 480) mosquitoes.

**Table 5:** Estimated marginal means of swarm size following the month.

Month		Mean	Standard error	95% Wald Confidence Interval	
	N			Lower	Upper
13.11	34	260.91 a	30.92	206.84	329.12
14.03	33	225.18 a	23.79	183.07	276.98
13.10	68	157.87ab	18.79	125.01	199.35
13.12	62	155.18 ab	16.98	125.22	192.29
14.01	38	104.74 bc	15.57	78.27	140.15
14.05	37	98.73 bcd	21.38	64.58	150.95
14.02	35	97.46 bc	13.22	74.71	127.13
14.06	35	93.77 bcd	18.67	63.47	138.53
13.07	33	72.3 cd	8.02	58.18	89.86
14.04	41	42.29 d	6.83	30.82	58.04
13.09	32	36.5 d	5.39	27.32	48.76
13.08	32	19.78 d	2.51	15.42	25.37

Different letters indicate statistically significant differences between groups (Sequential Bonferroni pairwise comparisons; the mean difference is significant at the 0.05 level).

Mating pairs were rarely found in April, June, or July and none was observed in August or September (Fig 2C). In total, 10,550 mating pairs were observed, with up to 287 per swarm and a mean of 118.55±6.05. A significant difference in numbers of mating pairs per swarm was found based on the month of observation (GLNM, df = 11, Wald χ^2^ = 95.123, *P*<0.001, Table 6). The time between swarm formation and the first observed mating by month is shown in Fig 2D. Time to first mating varied between 2 and 35 minutes with a mean of 13.32 ± 0.37 (N = 310) and was significantly different between months (GLNM, df = 9, Wald χ^2^ = 237.597, *P*<0.001, Table 7). The shortest times were mostly recorded in October and the longest in January.

**Table 6:** Estimated marginal means of mating pairs per swarm following the month.

Month		Mean	Standard error	95% Wald Confidence Interval	
	N			Lower	Upper
14.03	33	79.97 a	12.32	59.13	108.16
13.11	34	45.88 ab	5.89	35.67	59.01
14.02	35	30.23 bc	5.63	20.99	43.54
13.12	62	27.31 bcd	5.18	18.83	39.61
14.01	38	23.53 bcde	5.84	14.46	38.28
14.05	37	20.08 cde	4.96	12.38	32.57
13.10	60	19.38 cd	3.77	13.24	28.37
14.06	25	13.76 cdef	5.08	6.67	28.38
14.04	37	8.03 def	3.32	3.57	18.07
13.07	33	4.82 ef	1.90	2.22	10.45
13.09	32	0.00 f	0.00	0.00	0.00
13.08	32	0.00 f	0.00	0.00	0.00

Different letters indicate statistically significant differences between groups (Sequential Bonferroni pairwise comparisons; the mean difference is significant at the 0.05 level).

**Table 7:** Estimated marginal means of time to first mating following the month.

Month		Mean	Standard error	95% Wald Confidence Interval	
	N			Lower	Upper
14.01	37	21.49 a	0.76	20.04	23.03
13.12	58	16.34 b	0.81	14.84	18
14.02	33	14.61 bc	0.96	12.85	16.6
14.04	16	13.00 bcd	0.86	11.42	14.8
14.06	20	11.75 cde	1.22	9.58	14.41
13.07	13	11.23 cde	1.09	9.28	13.59
14.05	26	11.23 cde	0.69	9.96	12.66
14.03	31	10.84 de	0.97	9.1	12.9
13.11	34	10.38 de	0.85	8.84	12.2
13.10	42	7.98 e	0.67	6.77	9.39

### The relationship between *An*. *arabiensis* swarming and mating parameters and time of sunset in Dioulassoba

The relationship between the swarming and mating parameters and time of sunset (Pearson’s correlation test with significance at the 0.05 level (2-tailed)) is shown in Table 8. A strong positive correlation was found between the time of sunset and both start and end times. Moderate positive correlations were observed between swarming duration and both the swarm size and the number of mating pairs. However, a strong positive correlation was found between the number of mating pairs and the swarm size. In addition, the later the first mating occurred, the longer the swarming lasted.

**Table 8:** Correlation between swarming, mating parameters and sunset.

		Sunset	Start time	End time	Duration	Swarm size	Mating pairs per swarm	Time to 1^st^ mating pair
**Sunset**	**r**	1	.934**	.980**	-.280**	-.339**	-.146**	-.011
	**Sig.**		.000	.000	.000	.000	.002	.842
	**N**	480	480	480	480	480	458	310
**Start time**	**r**	.934**	1	.927**	-.574**	-.405**	-.244**	-.258**
	**Sig.**	.000		.000	.000	.000	.000	.000
	**N**	480	480	480	480	480	458	310
**End time**	**r**	.980**	.927**	1	-.224**	-.287**	-.097*	-.036
	**Sig.**	.000	.000		.000	.000	.038	.528
	**N**	480	480	480	480	480	458	310
**Duration**	**r**	-.286**	-.574**	-.224**	1	.426**	.421**	.622**
	**Sig.**	.000	.000	.000		.000	.000	.000
	**N**	480	480	480	480	480	458	310
**Density**	**r**	-.339**	-.405**	-.287**	.426**	1	.747**	-.158**
	**Sig.**	.000	.000	.000	.000		.000	.005
	**N**	480	480	480	480	480	458	310
**mating pairs per swarm**	**r**	-.146**	-.244**	-.097*	.421**	.747**	1	-.170**
	**Sig.**	.002	.000	.038	.000	.000		.003
	**N**	458	458	458	458	458	458	301
**Time 1**^**st**^ **mating pair**	**r**	-.011	-.258**	-.036	.622**	-.158**	-.170**	1
	**Sig.**	.842	.000	.528	.000	.005	.003	
	**N**	310	310	310	310	310	301	310

r = Pearson correlation; Number of values; Sig.: Significance

**. Correlation is significant at the 0.01 level (2-tailed)

*. Correlation is significant at the 0.05 level (2-tailed).

### The relationship between *An*. *arabiensis* swarming and mating parameters and the monthly temperature, rainfall, rain frequency and time of sunshine in Dioulassoba

**Fig 3:**
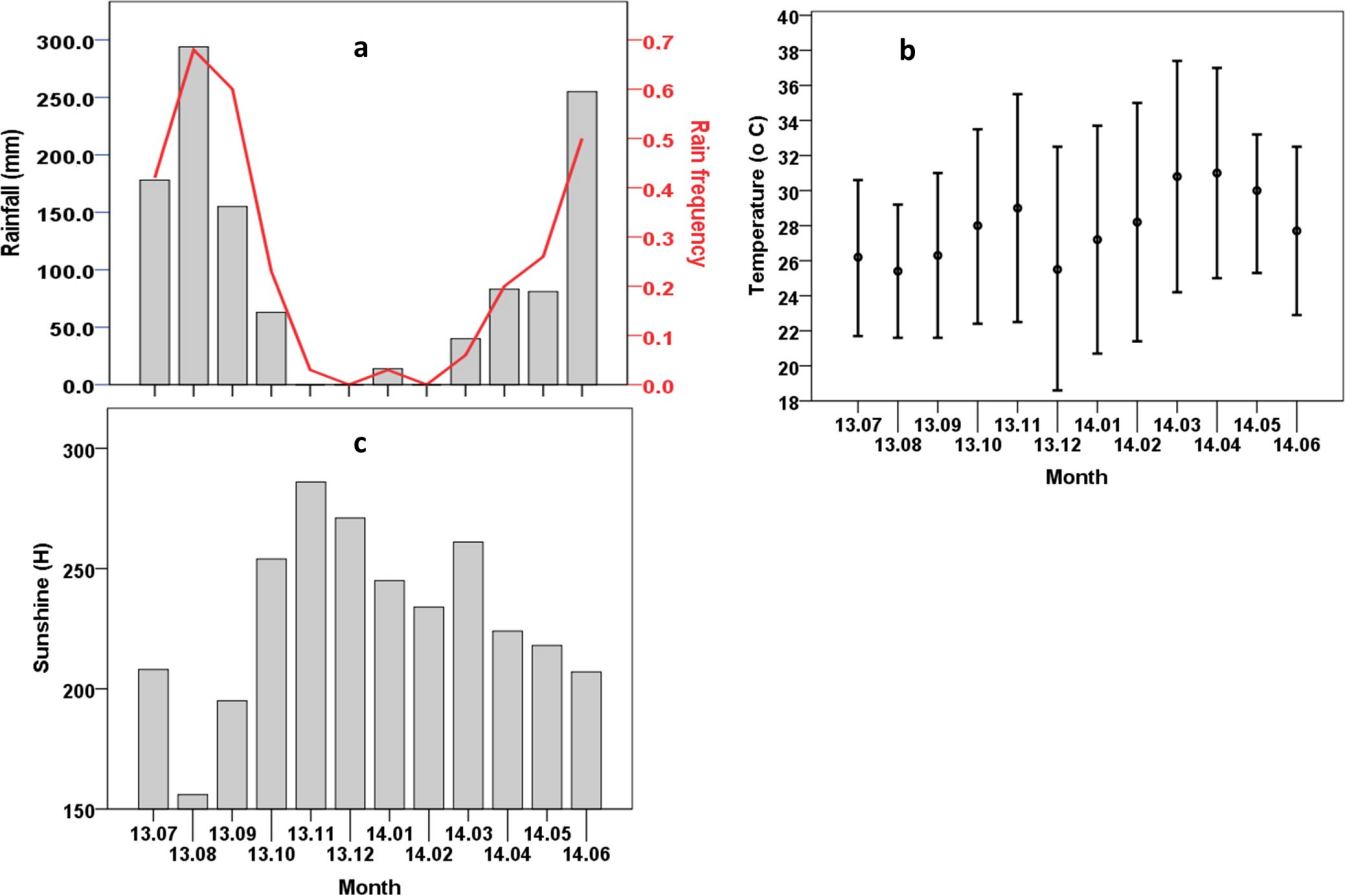
Monthly climatic conditions in Dioulassoba over a year. (a) Rainfall (histogram) and rain frequency (red line). (b) Minimal, mean and maximal temperature, respectively at the bottom, middle and top of the bar. (c) Duration of sunshine.

The monthly rainfall and rain frequency were similarly variable (Fig 3A). Both were in an inverse relationship with the swarming and the mating dynamics: the higher the rainfall and the rain frequency were, the lower were the swarming duration, swarm size and especially the number of mating pairs per swarm. In the same way, a negative correlation was observed between relative humidity at the start and end of swarming and the swarm size, duration and the number of mating-pairs per swarm (Table 9). The variation in monthly hours of sunshine (Fig 3C) followed an inverse trend of that of the rainfall, and the rain frequency and was thus in a positive relationship with swarming and mating parameters. However, the variation in temperature (minimum, mean and maximum) (Fig 3B) did not correlate with the swarming and mating dynamics. Although March and April had similarly high temperatures, the swarming parameters were found to be significantly different between these months. The temperatures at the start and end of swarming were moderately correlated with the swarming start and end times themselves (Table 9).

**Table 9:** Correlation between swarming, mating parameters and, temperature and relative humidity.

		Sunset	Start time	End time	Duration	Swarm size	Mating pairs per swarm	Time to 1^st^ mating pair
**Temperature (start time)**	**r**	-.286**	-.437**	-.272**	.452**	.291**	.267**	.052
	**Sig.**	.000	.000	.000	.000	.000	.000	.540
	**N**	231	231	231	231	231	220	140
**Temperature (end time)**	**r**	-.297**	-.356**	-.273**	.284**	.283**	.229**	-.294**
	**Sig.**	.000	.000	.000	.000	.000	.001	.000
	**N**	231	231	231	231	231	220	140
**RH start time**	**r**	.369**	.614**	.339**	-.705**	-.413**	-.347**	-.441**
	**Sig.**	.000	.000	.000	.000	.000	.000	.000
	**N**	231	231	231	231	231	220	140
**RH (end time)**	**r**	.284**	.546**	.266**	-.684**	-.391**	-.340**	-.306**
	**Sig.**	.000	.000	.000	.000	.000	.000	.000
	**N**	231	231	231	231	231	220	140

RH: Relative humidity

r = Pearson correlation; Number of values; Sig.: Significance

**. Correlation is significant at the 0.01 level (2-tailed)

*. Correlation is significant at the 0.05 level (2-tailed).

### Characterization of mosquitoes collected from swarms: female insemination rate, *Plasmodium falciparum* infection rate, wing length, proportion of freshly sugar-fed males

In total, 2,316 mating pairs were collected. Sixty eight (2.97%) out of the 2,316 females caught *in copula* were blood fed. Out of 1,439 females analyzed, 1,417 (98.47%) were inseminated. None of the 506 females tested was found to carry *P*. *falciparum*.

**Fig 4:**
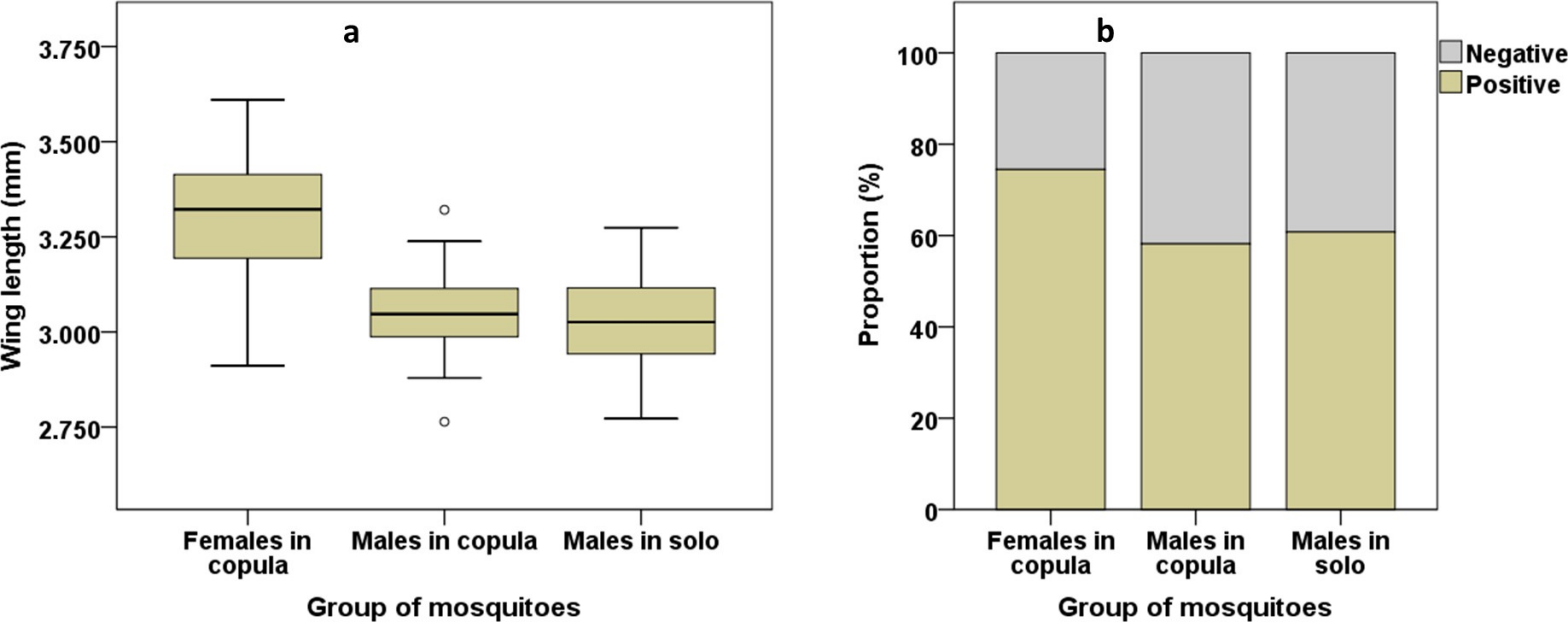
Characteristics of *An*. *arabiensis* males and females collected from swarms in Dioulassoba. (a) Wing length of males *in solo*, *in copula* and females *in copula*. (b) Proportions of males *in solo*, *in copula* and females *in copula* containing a recent sugar meal.

The wing lengths of mosquitoes collected from swarms are summarized in Fig 4A. The size range of males collected *in solo* (3.0281 ± 0.0155 mm, N = 55) was slightly smaller than that of males collected *in copula* (3.0506 ± 0.0136 mm, N = 55) though not significantly so (P = 0.275, Sequential Bonferroni pairwise comparison). Females collected *in copula* (3.2973 ± 0.0248 mm, N = 55) had higher size than both groups of males (P<0.001, Sequential Bonferroni pairwise comparison). Overall, more than 50% of males, collected either *in copula* or *in solo*, and more than 70% of females caught from swarms were positive in the Cold-Anthrone Test, indicating the presence of fresh sugar solution in their abdomens (Fig 4B). This proportion was similar in males collected *in copula* (58.16% (265), N = 456) and *in solo* (60.78% (279), N = 459) (P = 0.4198, Fisher’s exact test). Both proportions were lower than that of females caught *in copula* (74.45% (314), N = 423) (P<0.05, Fisher’s exact tests).

## Discussion

The present study reports on the field parameters which supported the occurrence of *Anopheles arabiensis* swarms over the course of a year in Dioulassoba, a district of Bobo-Dioulasso, Burkina Faso. The observation of swarms throughout the whole year highlights the ability of *An*. *arabiensis* to swarm in a large range of climatic conditions, including sunset time, temperature, and days and levels of precipitation, because these factors varied significantly between months. However, swarming and mating dynamics were affected by environmental factors. In this country, two seasons take place, namely the rainy season from April to October and the dry season from November to March. Swarms of *An*. *arabiensis* were found each month, corroborating the observations of Dabiré et al. [27] who targeted periods in September, October, March and April for swarm observations. However, parameters including the start and end times of swarming, swarm duration, and the number of mosquitoes and mating pairs per swarm varied according to the month and the season. The start and end times of swarming decreased from July to December and increased from December to July. The strong correlation between the start and end times of swarming and the time of sunset corroborates previous reports on *Anopheles* species, which is already known to start swarming after sunset [11]. However, here we found that the relationship between onset of swarming and time of sunset varied throughout the year; from late July to early October the swarms mostly appeared after or at sunset, but more often before sunset during the other part of the year.

The duration of swarming and the number of mosquitoes per swarm were found to be in an inverse relationship with total rainfall and the regularity of rain. The shortest swarm times and smallest swarms were observed during the peak of the rainy season, especially in August and September, and swarms were larger and lasted longer during the dry season. The durations recorded during the dry season were up to the twice those observed during the rainy season and those reported commonly by previous studies on *Anopheles* species [32]. Knowing that mosquito flight is energy-dependent [33, 34], males that join the swarms during the dry season would need to have more substantial reserves in comparison to their counterparts collected in the rainy season.

As with many Diptera [7, 10, 35], *An*. *gambiae s*. *l*. [10] mating occurs mostly in swarms, and our study reveals that *An*. *arabiensis* reproduction would seem to be more intensive during the dry than the rainy season in the study area. Indeed, no mating pairs were observed during the period of the peak rainfall, August and September. This result, together with the variation in swarm size across the seasons, showed seasonal fluctuations in the dynamics of swarming and mating of *An*. *arabiensis* in Dioulassoba. The causes of this phenomenon, as well as the survival strategies of this species in this area at different times of the year, need to be more deeply investigated; our study was observational and could not provide causal explanations. However, regarding the relationship between variations in the climatic factors and swarming and mating dynamics, where we observed a lower level of reproductive activity during the peak of the rainy season, we would propose the following as possible hypotheses. *i*) The frequent rains physically hindered swarm formation and an alternative mating strategy would have to have been employed, such as mating indoor resting sites. In this case, the population density would not be affected by the seasons. However, some reports on its seasonal density in the same area (Dioulassoba) have stated that this species was observed in higher numbers during the dry season [25, 26, Bimbilé Somda *et al*., unpublished]. *ii*) The high precipitations and, related high relative humidity, were unfavourable to swarming and/or mating, leading to lower reproductive activity in this period. In this case, a migration-recolonization model could be an alternative explanation for the increase in population in the dry season. However, the mosquito population cannot build up immediately when then rains became scarce, whereas a rapid increase was observed in this study. *iii*) The Houet river was the main larval breeding site and was drained of larvae by the high rainfall and regular flooding of the rainy season; the residual population was then able to increase to a high population level when suitable larval sites were available. *iiii*) A combination of different hypotheses is possible. The impact of hydrography and topography on the water flow and the formation of water pools suitable for mosquito breeding are fairly well understood [36, 37]. Our results are consistent with the observations of Peixoto in the Amazon region where the water level of the rivers increases dramatically during the rainy season, flooding the areas immediately proximal to the margins and when the rainy season ends the water level decreases, and pools of water suitable for mosquito breeding appear because of the irregularity of the rain (in [38]). This may explain the increase in swarm size and mating pairs starting in October which coincides with the end of the rainy season in Dioulassoba, and conversely, those in April which marks the rainy season onset. Assuming a lower reproductive success in the period of frequent rains, a direct destruction of swarms in this period would consequently reduce the density of this species, as assumed by Sawadogo *et al*. [39] for *An*. *gambiae*. Although targeting large swarms would be more effective, the targeted implementation of the SIT would also be effective, releasing large numbers of sterile males when the population is at its lowest to more readily achieve an over-flooding ratio. Further investigations are needed to better elucidate the causes of lower reproductive activities in *An*. *arabiensis* and its strategies for population maintenance during the frequent rains in this area to better define a technique of control.

The low proportion of blood-fed females caught during copulation indicates that female *An*. *arabiensis* can bite before mating but would prefer to mate before seeking a blood meal. The lack of *P*. *falciparum*-infected females collected *in copula* suggests that young females join swarms to mate, as the parasite takes 2 to 3 weeks to be detectable as sporozoites in the mosquito salivary glands [40]. In contrast to the observation of Hassan et al. [41] in Sudan, in our study, almost all the females caught *in copula* were inseminated. This suggests a successful and fertile copulation in the field and must be considered in the development of biological control programs based on mating. Such techniques would have to provide both sufficient numbers and sufficiently competitive males to compete with the apparently successful wild males. All males collected from swarms, those *in copula* and those collected *in solo*, were similar in body size to those reported by previous studies on the *An*. *gambiae* complex [42]. This result indicates that the mating capability could not be predicted by body size. Adult mosquito life history traits are known to be determined by development conditions at the larval stage [43, 44], and since the Houet river which is likely to be the main larval site and would provide similar conditions for all larvae, adult characteristics including body size would be expected to be similar across the study site. Moreover, the similarity in terms of the proportion of males which had freshly fed on sugar of those collected *in copula* and those collected *in solo* indicates that males had equal success in entering and mating in a swarm whether they had sugar fed or not. The range of potential nectar sources present in the study site, such as *Thevetia neriifolia*, *Barleria lupilina*, *Lannea microcarpa* and *Mangifera indica* [45, 46], would provide ready access to sugar meal for mosquitoes before and/or after swarming.

## Conclusion

*An*. *arabiensis* is known to be well adapted to arid environments. Its colonization of the Sudanese region of Burkina Faso is assumed to be due to ecological adaptations from dry to more humid settings. However, we have discovered that even though the species is now consistently present in this new ecological area throughout the year, the optimal time for its reproductive activities is during the dry season. Because of this seasonal peak in reproduction, targeting interventions to kill mosquitoes in swarms during the rainfall peak (August and September) would be effective in reducing the population density of this vector. This approach would be more efficient than applying the intervention throughout the year, including times when swarms are not key to maintaining the population. More broadly, our findings on the ecology of reproduction of *An*. *arabiensis* should be considered in the development or implementation of any control strategy, especially those based on mating biology such as the sterile insect technique. However, further investigations are needed to better elucidate the causes of low reproductive activities and strategies of population maintenance during the period of frequent rains of this species in this area.

## Supporting information

S1 Table Dataset. (XLSX)

S1 File Summary of statistical analysis (GNLM) outputs. (PDF)
