# Supplementary material for: Ecology of reproduction of Anopheles arabiensis in an urban area of Bobo-Dioulasso, Burkina Faso (West Africa): Monthly swarming and mating frequency and their relation to environmental factors
Source: PLoS One. 2018 Nov 7;13(11):e0205966. doi: 10.1371/journal.pone.0205966 (PMC6221289; doi:10.1371/journal.pone.0205966)
Supplement: S1 File — (PDF) [file pone.0205966.s002.pdf]

Summary of statistical analysis (GLNM) outputs

- **Dependent Variable: Swarming start time; Predictor: Month**

Model Information

Dependent Variable	Start time
Probability Distribution	Gamma
Link Function	Log

Continuous Variable Information

		N	Minimum	Maximum	Mean	Std. Deviation
Dependent Variable	Start time	480	1047	1127	1091.10	19.954

Goodness of Fit[a]

	Value	df	Value/df
Deviance	.013	468	.000
Scaled Deviance	480.002	468	
Pearson Chi-Square	.013	468	.000
Scaled Pearson Chi-Square	480.108	468	
Log Likelihood[b]	-1516.651		
Akaike's Information Criterion (AIC)	3059.301		
Finite Sample Corrected AIC (AICC)	3060.083		
Bayesian Information Criterion (BIC)	3113.561		
Consistent AIC (CAIC)	3126.561		

Model: (Intercept), Month[a]
a. Information criteria are in smaller-is-better form.
b. The full log likelihood function is displayed and used in computing information criteria.

Omnibus Test[a]

Likelihood Ratio Chi-Square	df	Sig.
1202.158	11	.000

Model: (Intercept), Month[a]
a. Compares the fitted model against the intercept-only model.

Tests of Model Effects

Source	Type III

	Wald Chi-Square	df	Sig.
(Intercept)	936117893.492	1	.000
Month	8797.566	11	.000

Model: (Intercept), Month

Estimated Marginal Means: Month

Month	Mean	Std. Error	95% Wald Confidence Interval	
			Lower	Upper
13.07	1120.82	.458	1119.92	1121.72
13.08	1112.62	.679	1111.30	1113.96
13.09	1098.69	1.107	1096.52	1100.86
13.10	1076.41	.872	1074.70	1078.12
13.11	1065.24	.623	1064.02	1066.46
13.12	1065.24	.825	1063.63	1066.86
14.01	1073.95	1.319	1071.37	1076.54
14.02	1089.83	.816	1088.23	1091.43
14.03	1099.94	.935	1098.11	1101.77
14.04	1104.63	.613	1103.43	1105.84
14.05	1106.57	.584	1105.42	1107.71
14.06	1115.26	1.117	1113.07	1117.45

Pairwise Comparisons

(I) Month	(J) Month	Mean Difference (I-J)	Std. Error	df	Sequential Bonferroni Sig.	95% Wald Confidence Interval for Difference[b]	
						Lower	Upper
13.07	13.08	8.19[a]	.819	1	.000	5.44	10.95
	13.09	22.13[a]	1.198	1	.000	18.10	26.16
	13.10	44.41[a]	.985	1	.000	41.10	47.72
	13.11	55.58[a]	.773	1	.000	52.99	58.18
	13.12	55.58[a]	.943	1	.000	52.42	58.74
	14.01	46.87[a]	1.396	1	.000	42.20	51.54
	14.02	30.99[a]	.936	1	.000	27.86	34.12
	14.03	20.88[a]	1.041	1	.000	17.40	24.35
	14.04	16.18[a]	.765	1	.000	13.63	18.73
	14.05	14.25[a]	.743	1	.000	11.78	16.72
	14.06	5.56[a]	1.208	1	.000	2.31	8.81
13.08	13.07	-8.19[a]	.819	1	.000	-10.95	-5.44
	13.09	13.94[a]	1.298	1	.000	9.62	18.25
	13.10	36.21[a]	1.105	1	.000	32.55	39.88
	13.11	47.39[a]	.921	1	.000	44.34	50.44
	13.12	47.38[a]	1.068	1	.000	43.85	50.91
	14.01	38.68[a]	1.483	1	.000	33.78	43.57
	14.02	22.80[a]	1.061	1	.000	19.30	26.29

	14.03	12.69[a]	1.155	1	.000	8.88	16.49
	14.04	7.99[a]	.914	1	.000	4.99	10.99
	14.05	6.06[a]	.895	1	.000	3.45	8.67
	14.06	-2.63	1.307	1	.176	-5.90	.63
13.09	13.07	-22.13[a]	1.198	1	.000	-26.16	-18.10
	13.08	-13.94[a]	1.298	1	.000	-18.25	-9.62
	13.10	22.28[a]	1.409	1	.000	17.65	26.90
	13.11	33.45[a]	1.270	1	.000	29.30	37.61
	13.12	33.45[a]	1.380	1	.000	28.94	37.96
	14.01	24.74[a]	1.722	1	.000	19.13	30.35
	14.02	8.86[a]	1.375	1	.000	4.88	12.83
	14.03	-1.25	1.449	1	.775	-4.50	2.00
	14.04	-5.95[a]	1.265	1	.000	-9.41	-2.49
	14.05	-7.88[a]	1.252	1	.000	-11.47	-4.29
	14.06	-16.57[a]	1.573	1	.000	-21.69	-11.45
13.10	13.07	-44.41[a]	.985	1	.000	-47.72	-41.10
	13.08	-36.21[a]	1.105	1	.000	-39.88	-32.55
	13.09	-22.28[a]	1.409	1	.000	-26.90	-17.65
	13.11	11.18[a]	1.072	1	.000	7.70	14.66
	13.12	11.17[a]	1.200	1	.000	7.28	15.06
	14.01	2.46	1.581	1	.357	-1.32	6.25
	14.02	-13.42[a]	1.194	1	.000	-17.28	-9.55
	14.03	-23.53[a]	1.279	1	.000	-27.65	-19.40
	14.04	-28.22[a]	1.066	1	.000	-31.65	-24.79
	14.05	-30.16[a]	1.050	1	.000	-33.53	-26.78
	14.06	-38.85[a]	1.417	1	.000	-43.39	-34.30
13.11	13.07	-55.58[a]	.773	1	.000	-58.18	-52.99
	13.08	-47.39[a]	.921	1	.000	-50.44	-44.34
	13.09	-33.45[a]	1.270	1	.000	-37.61	-29.30
	13.10	-11.18[a]	1.072	1	.000	-14.66	-7.70
	13.12	-.01	1.033	1	.995	-2.03	2.02
	14.01	-8.71[a]	1.458	1	.000	-12.81	-4.62
	14.02	-24.59[a]	1.026	1	.000	-27.87	-21.31
	14.03	-34.70[a]	1.123	1	.000	-38.29	-31.12
	14.04	-39.40[a]	.874	1	.000	-42.18	-36.62
	14.05	-41.33[a]	.854	1	.000	-44.04	-38.62
	14.06	-50.02[a]	1.279	1	.000	-54.07	-45.98
13.12	13.07	-55.58[a]	.943	1	.000	-58.74	-52.42
	13.08	-47.38[a]	1.068	1	.000	-50.91	-43.85
	13.09	-33.45[a]	1.380	1	.000	-37.96	-28.94
	13.10	-11.17[a]	1.200	1	.000	-15.06	-7.28
	13.11	.01	1.033	1	.995	-2.02	2.03
	14.01	-8.71[a]	1.555	1	.000	-13.02	-4.39
	14.02	-24.59[a]	1.160	1	.000	-28.24	-20.93
	14.03	-34.70[a]	1.247	1	.000	-38.62	-30.78
	14.04	-39.39[a]	1.027	1	.000	-42.61	-36.17
	14.05	-41.33[a]	1.010	1	.000	-44.48	-38.17

	14.06	-50.02[a]	1.388	1	.000	-54.34	-45.69
14.01	13.07	-46.87[a]	1.396	1	.000	-51.54	-42.20
	13.08	-38.68[a]	1.483	1	.000	-43.57	-33.78
	13.09	-24.74[a]	1.722	1	.000	-30.35	-19.13
	13.10	-2.46	1.581	1	.357	-6.25	1.32
	13.11	8.71[a]	1.458	1	.000	4.62	12.81
	13.12	8.71[a]	1.555	1	.000	4.39	13.02
	14.02	-15.88[a]	1.551	1	.000	-20.69	-11.07
	14.03	-25.99[a]	1.617	1	.000	-30.99	-21.00
	14.04	-30.69[a]	1.454	1	.000	-35.16	-26.21
	14.05	-32.62[a]	1.442	1	.000	-37.04	-28.20
	14.06	-41.31[a]	1.728	1	.000	-46.59	-36.03
14.02	13.07	-30.99[a]	.936	1	.000	-34.12	-27.86
	13.08	-22.80[a]	1.061	1	.000	-26.29	-19.30
	13.09	-8.86[a]	1.375	1	.000	-12.83	-4.88
	13.10	13.42[a]	1.194	1	.000	9.55	17.28
	13.11	24.59[a]	1.026	1	.000	21.31	27.87
	13.12	24.59[a]	1.160	1	.000	20.93	28.24
	14.01	15.88[a]	1.551	1	.000	11.07	20.69
	14.03	-10.11[a]	1.241	1	.000	-13.78	-6.44
	14.04	-14.81[a]	1.020	1	.000	-17.91	-11.71
	14.05	-16.74[a]	1.003	1	.000	-19.77	-13.71
	14.06	-25.43[a]	1.383	1	.000	-29.59	-21.27
14.03	13.07	-20.88[a]	1.041	1	.000	-24.35	-17.40
	13.08	-12.69[a]	1.155	1	.000	-16.49	-8.88
	13.09	1.25	1.449	1	.775	-2.00	4.50
	13.10	23.53[a]	1.279	1	.000	19.40	27.65
	13.11	34.70[a]	1.123	1	.000	31.12	38.29
	13.12	34.70[a]	1.247	1	.000	30.78	38.62
	14.01	25.99[a]	1.617	1	.000	21.00	30.99
	14.02	10.11[a]	1.241	1	.000	6.44	13.78
	14.04	-4.69[a]	1.118	1	.000	-7.64	-1.75
	14.05	-6.63[a]	1.102	1	.000	-9.76	-3.50
	14.06	-15.32[a]	1.457	1	.000	-19.68	-10.96
14.04	13.07	-16.18[a]	.765	1	.000	-18.73	-13.63
	13.08	-7.99[a]	.914	1	.000	-10.99	-4.99
	13.09	5.95[a]	1.265	1	.000	2.49	9.41
	13.10	28.22[a]	1.066	1	.000	24.79	31.65
	13.11	39.40[a]	.874	1	.000	36.62	42.18
	13.12	39.39[a]	1.027	1	.000	36.17	42.61
	14.01	30.69[a]	1.454	1	.000	26.21	35.16
	14.02	14.81[a]	1.020	1	.000	11.71	17.91
	14.03	4.69[a]	1.118	1	.000	1.75	7.64
	14.05	-1.93	.847	1	.112	-4.11	.25
	14.06	-10.62[a]	1.274	1	.000	-14.41	-6.83
14.05	13.07	-14.25[a]	.743	1	.000	-16.72	-11.78
	13.08	-6.06[a]	.895	1	.000	-8.67	-3.45

	13.09	7.88[a]	1.252	1	.000	4.29	11.47
	13.10	30.16[a]	1.050	1	.000	26.78	33.53
	13.11	41.33[a]	.854	1	.000	38.62	44.04
	13.12	41.33[a]	1.010	1	.000	38.17	44.48
	14.01	32.62[a]	1.442	1	.000	28.20	37.04
	14.02	16.74[a]	1.003	1	.000	13.71	19.77
	14.03	6.63[a]	1.102	1	.000	3.50	9.76
	14.04	1.93	.847	1	.112	-.25	4.11
	14.06	-8.69[a]	1.261	1	.000	-12.39	-4.99
14.06	13.07	-5.56[a]	1.208	1	.000	-8.81	-2.31
	13.08	2.63	1.307	1	.176	-.63	5.90
	13.09	16.57[a]	1.573	1	.000	11.45	21.69
	13.10	38.85[a]	1.417	1	.000	34.30	43.39
	13.11	50.02[a]	1.279	1	.000	45.98	54.07
	13.12	50.02[a]	1.388	1	.000	45.69	54.34
	14.01	41.31[a]	1.728	1	.000	36.03	46.59
	14.02	25.43[a]	1.383	1	.000	21.27	29.59
	14.03	15.32[a]	1.457	1	.000	10.96	19.68
	14.04	10.62[a]	1.274	1	.000	6.83	14.41
	14.05	8.69[a]	1.261	1	.000	4.99	12.39

Pairwise comparisons of estimated marginal means based on the original scale of dependent variable Start time

a. The mean difference is significant at the .05 level.

b. Confidence interval bounds are approximate.

- **Dependent Variable: Swarming end time; Predictor: Month**

Model Information

Dependent Variable	End time
Probability Distribution	Gamma
Link Function	Log

Continuous Variable Information

		N	Minimum	Maximum	Mean	Std. Deviation
Dependent Variable	Endtime	480	1090	1147	1119.41	16.767

Goodness of Fit[a]

	Value	df	Value/df
Deviance	.006	468	.000
Scaled Deviance	480.002	468	
Pearson Chi-Square	.006	468	.000
Scaled Pearson Chi-Square	479.765	468	
Log Likelihood[b]	-1345.691		

Akaike's Information Criterion (AIC)	2717.382		
Finite Sample Corrected AIC (AICC)	2718.163		
Bayesian Information Criterion (BIC)	2771.641		
Consistent AIC (CAIC)	2784.641		

Model: (Intercept), Month[a]
a. Information criteria are in smaller-is-better form.
b. The full log likelihood function is displayed and used in computing information criteria.

Omnibus Test[a]

Likelihood Ratio Chi-Square	df	Sig.
1376.749	11	.000

Model: (Intercept), Month[a]
a. Compares the fitted model against the intercept-only model.

Tests of Model Effects

Source	Type III		
	Wald Chi-Square	df	Sig.
(Intercept)	1861483484.523	1	.000
Month	13830.380	11	.000

Model: (Intercept), Month

Estimated Marginal Means: Month

Month	Mean	Std. Error	95% Wald Confidence Interval	
			Lower	Upper
13.07	1143.61	.340	1142.94	1144.27
13.08	1132.47	.747	1131.00	1133.93
13.09	1119.44	.955	1117.57	1121.31
13.10	1101.43	.570	1100.31	1102.55
13.11	1095.53	.476	1094.60	1096.46
13.12	1100.05	.474	1099.12	1100.98
14.01	1113.05	.742	1111.60	1114.51
14.02	1123.14	.471	1122.22	1124.07
14.03	1130.55	.534	1129.50	1131.59
14.04	1128.59	.606	1127.40	1129.77
14.05	1134.35	.922	1132.55	1136.16
14.06	1143.20	.408	1142.40	1144.00

Pairwise Comparisons

(I) Month	(J) Month	Mean Difference (I-J)	Std. Error	df	Sequential Bonferroni Sig.	95% Wald Confidence Interval for Difference[b]	
						Lower	Upper
13.07	13.08	11.14[a]	.821	1	.000	8.37	13.90
	13.09	24.17[a]	1.014	1	.000	20.76	27.58
	13.10	42.18[a]	.664	1	.000	39.95	44.41
	13.11	48.08[a]	.585	1	.000	46.11	50.04
	13.12	43.56[a]	.583	1	.000	41.60	45.51
	14.01	30.55[a]	.817	1	.000	27.82	33.29
	14.02	20.46[a]	.581	1	.000	18.52	22.41
	14.03	13.06[a]	.633	1	.000	10.95	15.17
	14.04	15.02[a]	.695	1	.000	12.70	17.34
	14.05	9.25[a]	.983	1	.000	5.98	12.53
	14.06	.41	.531	1	.444	-.63	1.45
13.08	13.07	-11.14[a]	.821	1	.000	-13.90	-8.37
	13.09	13.03[a]	1.213	1	.000	9.00	17.06
	13.10	31.04[a]	.940	1	.000	27.92	34.16
	13.11	36.94[a]	.886	1	.000	34.00	39.88
	13.12	32.42[a]	.885	1	.000	29.49	35.35
	14.01	19.42[a]	1.053	1	.000	15.94	22.89
	14.02	9.33[a]	.884	1	.000	6.41	12.24
	14.03	1.92	.918	1	.145	-.37	4.22
	14.04	3.88[a]	.962	1	.000	1.25	6.51
	14.05	-1.88	1.187	1	.226	-4.54	.78
	14.06	-10.73[a]	.851	1	.000	-13.53	-7.93
13.09	13.07	-24.17[a]	1.014	1	.000	-27.58	-20.76
	13.08	-13.03[a]	1.213	1	.000	-17.06	-9.00
	13.10	18.01[a]	1.112	1	.000	14.36	21.67
	13.11	23.91[a]	1.067	1	.000	20.41	27.41
	13.12	19.39[a]	1.066	1	.000	15.90	22.88
	14.01	6.38[a]	1.210	1	.000	2.99	9.78
	14.02	-3.71[a]	1.065	1	.003	-6.52	-.90
	14.03	-11.11[a]	1.094	1	.000	-14.68	-7.53
	14.04	-9.15[a]	1.131	1	.000	-12.44	-5.85
	14.05	-14.91[a]	1.328	1	.000	-19.24	-10.58
	14.06	-23.76[a]	1.038	1	.000	-27.14	-20.38
13.10	13.07	-42.18[a]	.664	1	.000	-44.41	-39.95
	13.08	-31.04[a]	.940	1	.000	-34.16	-27.92
	13.09	-18.01[a]	1.112	1	.000	-21.67	-14.36
	13.11	5.90[a]	.743	1	.000	3.75	8.05
	13.12	1.38	.742	1	.189	-.40	3.15
	14.01	-11.63[a]	.936	1	.000	-14.67	-8.59
	14.02	-21.72[a]	.740	1	.000	-24.11	-19.32
	14.03	-29.12[a]	.781	1	.000	-31.65	-26.59
	14.04	-27.16[a]	.832	1	.000	-29.85	-24.47

	14.05	-32.92[a]	1.084	1	.000	-36.42	-29.43
	14.06	-41.77[a]	.701	1	.000	-44.03	-39.52
13.11	13.07	-48.08[a]	.585	1	.000	-50.04	-46.11
	13.08	-36.94[a]	.886	1	.000	-39.88	-34.00
	13.09	-23.91[a]	1.067	1	.000	-27.41	-20.41
	13.10	-5.90[a]	.743	1	.000	-8.05	-3.75
	13.12	-4.52[a]	.672	1	.000	-6.43	-2.61
	14.01	-17.52[a]	.882	1	.000	-20.35	-14.70
	14.02	-27.61[a]	.670	1	.000	-29.76	-25.47
	14.03	-35.02[a]	.715	1	.000	-37.30	-32.73
	14.04	-33.06[a]	.771	1	.000	-35.51	-30.60
	14.05	-38.82[a]	1.038	1	.000	-42.11	-35.53
	14.06	-47.67[a]	.627	1	.000	-49.65	-45.69
13.12	13.07	-43.56[a]	.583	1	.000	-45.51	-41.60
	13.08	-32.42[a]	.885	1	.000	-35.35	-29.49
	13.09	-19.39[a]	1.066	1	.000	-22.88	-15.90
	13.10	-1.38	.742	1	.189	-3.15	.40
	13.11	4.52[a]	.672	1	.000	2.61	6.43
	14.01	-13.00[a]	.881	1	.000	-15.78	-10.23
	14.02	-23.09[a]	.668	1	.000	-25.20	-20.99
	14.03	-30.50[a]	.714	1	.000	-32.73	-28.26
	14.04	-28.54[a]	.769	1	.000	-30.94	-26.13
	14.05	-34.30[a]	1.037	1	.000	-37.53	-31.08
	14.06	-43.15[a]	.625	1	.000	-45.09	-41.21
14.01	13.07	-30.55[a]	.817	1	.000	-33.29	-27.82
	13.08	-19.42[a]	1.053	1	.000	-22.89	-15.94
	13.09	-6.38[a]	1.210	1	.000	-9.78	-2.99
	13.10	11.63[a]	.936	1	.000	8.59	14.67
	13.11	17.52[a]	.882	1	.000	14.70	20.35
	13.12	13.00[a]	.881	1	.000	10.23	15.78
	14.02	-10.09[a]	.879	1	.000	-12.81	-7.37
	14.03	-17.49[a]	.914	1	.000	-20.31	-14.68
	14.04	-15.53[a]	.959	1	.000	-18.47	-12.59
	14.05	-21.30[a]	1.184	1	.000	-24.91	-17.69
	14.06	-30.15[a]	.847	1	.000	-32.72	-27.57
14.02	13.07	-20.46[a]	.581	1	.000	-22.41	-18.52
	13.08	-9.33[a]	.884	1	.000	-12.24	-6.41
	13.09	3.71[a]	1.065	1	.003	.90	6.52
	13.10	21.72[a]	.740	1	.000	19.32	24.11
	13.11	27.61[a]	.670	1	.000	25.47	29.76
	13.12	23.09[a]	.668	1	.000	20.99	25.20
	14.01	10.09[a]	.879	1	.000	7.37	12.81
	14.03	-7.40[a]	.712	1	.000	-9.56	-5.25
	14.04	-5.44[a]	.768	1	.000	-7.64	-3.24
	14.05	-11.21[a]	1.036	1	.000	-14.32	-8.09
	14.06	-20.06[a]	.623	1	.000	-21.92	-18.19
14.03	13.07	-13.06[a]	.633	1	.000	-15.17	-10.95

	13.08	-1.92	.918	1	.145	-4.22	.37
	13.09	11.11[a]	1.094	1	.000	7.53	14.68
	13.10	29.12[a]	.781	1	.000	26.59	31.65
	13.11	35.02[a]	.715	1	.000	32.73	37.30
	13.12	30.50[a]	.714	1	.000	28.26	32.73
	14.01	17.49[a]	.914	1	.000	14.68	20.31
	14.02	7.40[a]	.712	1	.000	5.25	9.56
	14.04	1.96	.808	1	.076	-.12	4.04
	14.05	-3.81[a]	1.066	1	.002	-6.67	-.94
	14.06	-12.65[a]	.672	1	.000	-14.65	-10.66
14.04	13.07	-15.02[a]	.695	1	.000	-17.34	-12.70
	13.08	-3.88[a]	.962	1	.000	-6.51	-1.25
	13.09	9.15[a]	1.131	1	.000	5.85	12.44
	13.10	27.16[a]	.832	1	.000	24.47	29.85
	13.11	33.06[a]	.771	1	.000	30.60	35.51
	13.12	28.54[a]	.769	1	.000	26.13	30.94
	14.01	15.53[a]	.959	1	.000	12.59	18.47
	14.02	5.44[a]	.768	1	.000	3.24	7.64
	14.03	-1.96	.808	1	.076	-4.04	.12
	14.05	-5.77[a]	1.104	1	.000	-8.83	-2.71
	14.06	-14.61[a]	.731	1	.000	-16.77	-12.46
14.05	13.07	-9.25[a]	.983	1	.000	-12.53	-5.98
	13.08	1.88	1.187	1	.226	-.78	4.54
	13.09	14.91[a]	1.328	1	.000	10.58	19.24
	13.10	32.92[a]	1.084	1	.000	29.43	36.42
	13.11	38.82[a]	1.038	1	.000	35.53	42.11
	13.12	34.30[a]	1.037	1	.000	31.08	37.53
	14.01	21.30[a]	1.184	1	.000	17.69	24.91
	14.02	11.21[a]	1.036	1	.000	8.09	14.32
	14.03	3.81[a]	1.066	1	.002	.94	6.67
	14.04	5.77[a]	1.104	1	.000	2.71	8.83
	14.06	-8.85[a]	1.008	1	.000	-11.81	-5.89
14.06	13.07	-.41	.531	1	.444	-1.45	.63
	13.08	10.73[a]	.851	1	.000	7.93	13.53
	13.09	23.76[a]	1.038	1	.000	20.38	27.14
	13.10	41.77[a]	.701	1	.000	39.52	44.03
	13.11	47.67[a]	.627	1	.000	45.69	49.65
	13.12	43.15[a]	.625	1	.000	41.21	45.09
	14.01	30.15[a]	.847	1	.000	27.57	32.72
	14.02	20.06[a]	.623	1	.000	18.19	21.92
	14.03	12.65[a]	.672	1	.000	10.66	14.65
	14.04	14.61[a]	.731	1	.000	12.46	16.77
	14.05	8.85[a]	1.008	1	.000	5.89	11.81

Pairwise comparisons of estimated marginal means based on the original scale of dependent variable End time

a. The mean difference is significant at the .05 level.

b. Confidence interval bounds are approximate.

- **Dependent Variable: Swarming sunset time; Predictor: Month**

Model Information

Dependent Variable	Sunset
Probability Distribution	Gamma
Link Function	Log

Continuous Variable Information

		N	Minimum	Maximum	Mean	Std. Deviation
Dependent Variable	Sunset	480	1069	1123	1096.26	17.334

Goodness of Fit[a]

	Value	df	Value/df
Deviance	.003	468	.000
Scaled Deviance	480.001	468	
Pearson Chi-Square	.003	468	.000
Scaled Pearson Chi-Square	480.267	468	
Log Likelihood[b]	-1199.308		
Akaike's Information Criterion (AIC)	2424.616		
Finite Sample Corrected AIC (AICC)	2425.398		
Bayesian Information Criterion (BIC)	2478.876		
Consistent AIC (CAIC)	2491.876		

Model: (Intercept). Month[a]
a. Information criteria are in smaller-is-better form.
b. The full log likelihood function is displayed and used in computing information criteria.

Omnibus Test[a]

Likelihood Ratio Chi-Square	df	Sig.
1701.890	11	.000

Model: (Intercept). Month[a]
a. Compares the fitted model against the intercept-only model.

Tests of Model Effects

Source	Type III

	Wald Chi-Square	df	Sig.
(Intercept)	3411035910.201	1	.000
Month	94596.970	11	.000

Model: (Intercept). Month

Estimated Marginal Means: Month

Month	Mean	Std. Error	95% Wald Confidence Interval	
			Lower	Upper
13.07	1121.76	.219	1121.33	1122.19
13.08	1111.81	.789	1110.27	1113.36
13.09	1094.75	.824	1093.14	1096.37
13.10	1077.19	.453	1076.30	1078.08
13.11	1069.88	.123	1069.64	1070.12
13.12	1075.97	.454	1075.08	1076.86
14.01	1091.74	.620	1090.52	1092.95
14.02	1102.14	.379	1101.40	1102.89
14.03	1106.27	.089	1106.10	1106.45
14.04	1107.20	.062	1107.07	1107.32
14.05	1111.14	.306	1110.54	1111.74
14.06	1119.09	.290	1118.52	1119.65

Pairwise Comparisons

(I) Month	(J) Month	Mean Difference (I-J)	Std. Error	df	Sequential Bonferroni Sig.	95% Wald Confidence Interval for Difference[b]	
						Lower	Upper
13.07	13.08	9.95[a]	.818	1	.000	7.19	12.70
	13.09	27.01[a]	.853	1	.000	24.14	29.88
	13.10	44.57[a]	.503	1	.000	42.88	46.26
	13.11	51.88[a]	.251	1	.000	51.03	52.72
	13.12	45.79[a]	.504	1	.000	44.10	47.48
	14.01	30.02[a]	.658	1	.000	27.82	32.22
	14.02	19.61[a]	.438	1	.000	18.15	21.08
	14.03	15.48[a]	.236	1	.000	14.70	16.27
	14.04	14.56[a]	.227	1	.000	13.81	15.32
	14.05	10.62[a]	.376	1	.000	9.37	11.87
	14.06	2.67[a]	.363	1	.000	1.71	3.63
13.08	13.07	-9.95[a]	.818	1	.000	-12.70	-7.19
	13.09	17.06[a]	1.141	1	.000	13.27	20.85
	13.10	34.62[a]	.909	1	.000	31.60	37.64
	13.11	41.93[a]	.798	1	.000	39.29	44.57
	13.12	35.84[a]	.910	1	.000	32.84	38.85
	14.01	20.08[a]	1.003	1	.000	16.76	23.39
	14.02	9.67[a]	.875	1	.000	6.79	12.55
	14.03	5.54[a]	.794	1	.000	3.50	7.58

	14.04	4.62[a]	.791	1	.000	2.64	6.59
	14.05	.68	.846	1	.423	-.98	2.34
	14.06	-7.27[a]	.840	1	.000	-10.04	-4.51
13.09	13.07	-27.01[a]	.853	1	.000	-29.88	-24.14
	13.08	-17.06[a]	1.141	1	.000	-20.85	-13.27
	13.10	17.56[a]	.941	1	.000	14.47	20.65
	13.11	24.87[a]	.834	1	.000	22.13	27.60
	13.12	18.78[a]	.941	1	.000	15.70	21.86
	14.01	3.01[a]	1.032	1	.010	.54	5.48
	14.02	-7.39[a]	.908	1	.000	-9.83	-4.95
	14.03	-11.52[a]	.829	1	.000	-14.23	-8.81
	14.04	-12.45[a]	.827	1	.000	-15.14	-9.75
	14.05	-16.39[a]	.879	1	.000	-19.25	-13.52
	14.06	-24.34[a]	.874	1	.000	-27.17	-21.50
13.10	13.07	-44.57[a]	.503	1	.000	-46.26	-42.88
	13.08	-34.62[a]	.909	1	.000	-37.64	-31.60
	13.09	-17.56[a]	.941	1	.000	-20.65	-14.47
	13.11	7.31[a]	.469	1	.000	5.79	8.83
	13.12	1.22	.641	1	.113	-.21	2.66
	14.01	-14.55[a]	.768	1	.000	-17.03	-12.06
	14.02	-24.95[a]	.591	1	.000	-26.86	-23.05
	14.03	-29.08[a]	.461	1	.000	-30.57	-27.60
	14.04	-30.00[a]	.457	1	.000	-31.47	-28.54
	14.05	-33.94[a]	.547	1	.000	-35.70	-32.19
	14.06	-41.89[a]	.538	1	.000	-43.61	-40.18
13.11	13.07	-51.88[a]	.251	1	.000	-52.72	-51.03
	13.08	-41.93[a]	.798	1	.000	-44.57	-39.29
	13.09	-24.87[a]	.834	1	.000	-27.60	-22.13
	13.10	-7.31[a]	.469	1	.000	-8.83	-5.79
	13.12	-6.09[a]	.470	1	.000	-7.59	-4.59
	14.01	-21.85[a]	.632	1	.000	-23.87	-19.84
	14.02	-32.26[a]	.399	1	.000	-33.53	-31.00
	14.03	-36.39[a]	.152	1	.000	-36.87	-35.91
	14.04	-37.31[a]	.138	1	.000	-37.75	-36.88
	14.05	-41.25[a]	.330	1	.000	-42.29	-40.22
	14.06	-49.20[a]	.315	1	.000	-50.19	-48.22
13.12	13.07	-45.79[a]	.504	1	.000	-47.48	-44.10
	13.08	-35.84[a]	.910	1	.000	-38.85	-32.84
	13.09	-18.78[a]	.941	1	.000	-21.86	-15.70
	13.10	-1.22	.641	1	.113	-2.66	.21
	13.11	6.09[a]	.470	1	.000	4.59	7.59
	14.01	-15.77[a]	.769	1	.000	-18.17	-13.37
	14.02	-26.18[a]	.592	1	.000	-28.02	-24.33
	14.03	-30.30[a]	.462	1	.000	-31.74	-28.87
	14.04	-31.23[a]	.458	1	.000	-32.64	-29.81
	14.05	-35.17[a]	.547	1	.000	-36.85	-33.48
	14.06	-43.12[a]	.538	1	.000	-44.77	-41.47

14.01	13.07	-30.02[a]	.658	1	.000	-32.22	-27.82
	13.08	-20.08[a]	1.003	1	.000	-23.39	-16.76
	13.09	-3.01[a]	1.032	1	.010	-5.48	-.54
	13.10	14.55[a]	.768	1	.000	12.06	17.03
	13.11	21.85[a]	.632	1	.000	19.84	23.87
	13.12	15.77[a]	.769	1	.000	13.37	18.17
	14.02	-10.41[a]	.727	1	.000	-12.62	-8.19
	14.03	-14.54[a]	.626	1	.000	-16.44	-12.63
	14.04	-15.46[a]	.623	1	.000	-17.34	-13.57
	14.05	-19.40[a]	.692	1	.000	-21.48	-17.32
	14.06	-27.35[a]	.684	1	.000	-29.40	-25.30
14.02	13.07	-19.61[a]	.438	1	.000	-21.08	-18.15
	13.08	-9.67[a]	.875	1	.000	-12.55	-6.79
	13.09	7.39[a]	.908	1	.000	4.95	9.83
	13.10	24.95[a]	.591	1	.000	23.05	26.86
	13.11	32.26[a]	.399	1	.000	31.00	33.53
	13.12	26.18[a]	.592	1	.000	24.33	28.02
	14.01	10.41[a]	.727	1	.000	8.19	12.62
	14.03	-4.13[a]	.390	1	.000	-5.29	-2.97
	14.04	-5.05[a]	.384	1	.000	-6.19	-3.92
	14.05	-8.99[a]	.487	1	.000	-10.42	-7.56
	14.06	-16.94[a]	.477	1	.000	-18.33	-15.55
14.03	13.07	-15.48[a]	.236	1	.000	-16.27	-14.70
	13.08	-5.54[a]	.794	1	.000	-7.58	-3.50
	13.09	11.52[a]	.829	1	.000	8.81	14.23
	13.10	29.08[a]	.461	1	.000	27.60	30.57
	13.11	36.39[a]	.152	1	.000	35.91	36.87
	13.12	30.30[a]	.462	1	.000	28.87	31.74
	14.01	14.54[a]	.626	1	.000	12.63	16.44
	14.02	4.13[a]	.390	1	.000	2.97	5.29
	14.04	-.92[a]	.108	1	.000	-1.23	-.61
	14.05	-4.86[a]	.319	1	.000	-5.78	-3.95
	14.06	-12.81[a]	.303	1	.000	-13.67	-11.95
14.04	13.07	-14.56[a]	.227	1	.000	-15.32	-13.81
	13.08	-4.62[a]	.791	1	.000	-6.59	-2.64
	13.09	12.45[a]	.827	1	.000	9.75	15.14
	13.10	30.00[a]	.457	1	.000	28.54	31.47
	13.11	37.31[a]	.138	1	.000	36.88	37.75
	13.12	31.23[a]	.458	1	.000	29.81	32.64
	14.01	15.46[a]	.623	1	.000	13.57	17.34
	14.02	5.05[a]	.384	1	.000	3.92	6.19
	14.03	.92[a]	.108	1	.000	.61	1.23
	14.05	-3.94[a]	.312	1	.000	-4.82	-3.06
	14.06	-11.89[a]	.296	1	.000	-12.71	-11.07
14.05	13.07	-10.62[a]	.376	1	.000	-11.87	-9.37
	13.08	-.68	.846	1	.423	-2.34	.98
	13.09	16.39[a]	.879	1	.000	13.52	19.25

	13.10	33.94[a]	.547	1	.000	32.19	35.70
	13.11	41.25[a]	.330	1	.000	40.22	42.29
	13.12	35.17[a]	.547	1	.000	33.48	36.85
	14.01	19.40[a]	.692	1	.000	17.32	21.48
	14.02	8.99[a]	.487	1	.000	7.56	10.42
	14.03	4.86[a]	.319	1	.000	3.95	5.78
	14.04	3.94[a]	.312	1	.000	3.06	4.82
	14.06	-7.95[a]	.421	1	.000	-9.10	-6.80
14.06	13.07	-2.67[a]	.363	1	.000	-3.63	-1.71
	13.08	7.27[a]	.840	1	.000	4.51	10.04
	13.09	24.34[a]	.874	1	.000	21.50	27.17
	13.10	41.89[a]	.538	1	.000	40.18	43.61
	13.11	49.20[a]	.315	1	.000	48.22	50.19
	13.12	43.12[a]	.538	1	.000	41.47	44.77
	14.01	27.35[a]	.684	1	.000	25.30	29.40
	14.02	16.94[a]	.477	1	.000	15.55	18.33
	14.03	12.81[a]	.303	1	.000	11.95	13.67
	14.04	11.89[a]	.296	1	.000	11.07	12.71
	14.05	7.95[a]	.421	1	.000	6.80	9.10

Pairwise comparisons of estimated marginal means based on the original scale of dependent variable Sunset
a. The mean difference is significant at the .05 level.
b. Confidence interval bounds are approximate.

- **Dependent Variable: Swarming duration; Predictor: Month**

Model Information

Dependent Variable	Duration
Probability Distribution	Gamma
Link Function	Log

Continuous Variable Information

		N	Minimum	Maximum	Mean	Std. Deviation
Dependent Variable	Duration	480	13	53	28.31	7.696

Goodness of Fit[a]

	Value	df	Value/df
Deviance	17.446	468	.037
Scaled Deviance	482.890	468	
Pearson Chi-Square	16.243	468	.035
Scaled Pearson Chi-Square	449.591	468	
Log Likelihood[b]	-1473.801		

Akaike's Information Criterion (AIC)	2973.603		
Finite Sample Corrected AIC (AICC)	2974.384		
Bayesian Information Criterion (BIC)	3027.862		
Consistent AIC (CAIC)	3040.862		

Model: (Intercept). Month[a]
a. Information criteria are in smaller-is-better form.
b. The full log likelihood function is displayed and used in computing information criteria.

Omnibus Test[a]

Likelihood Ratio Chi-Square	df	Sig.
350.818	11	.000

Model: (Intercept). Month[a]
a. Compares the fitted model against the intercept-only model.

Tests of Model Effects

Source	Type III		
	Wald Chi-Square	df	Sig.
(Intercept)	155202.215	1	.000
Month	699.924	11	.000

Model: (Intercept). Month

Estimated Marginal Means: Month

Month	Mean	Std. Error	95% Wald Confidence Interval	
			Lower	Upper
13.07	22.79	.573	21.69	23.94
13.08	19.84	.460	18.96	20.77
13.09	20.75	.645	19.52	22.05
13.10	25.01	.642	23.79	26.30
13.11	30.29	.797	28.77	31.90
13.12	34.81	.801	33.27	36.41
14.01	39.11	1.101	37.01	41.32
14.02	33.31	.917	31.57	35.16
14.03	30.61	1.141	28.45	32.93
14.04	23.95	.773	22.48	25.51
14.05	27.78	.820	26.22	29.44
14.06	27.94	1.013	26.03	30.00

Pairwise Comparisons

(I) Month	(J) Month	Mean Difference (I-J)	Std. Error	df	Sequential Bonferroni Sig.	95% Wald Confidence Interval for Difference[b]	
						Lower	Upper
13.07	13.08	2.94[a]	.734	1	.001	.68	5.20
	13.09	2.04	.863	1	.218	-.43	4.51
	13.10	-2.23	.860	1	.144	-4.75	.30
	13.11	-7.51[a]	.982	1	.000	-10.72	-4.29
	13.12	-12.02[a]	.985	1	.000	-15.34	-8.70
	14.01	-16.32[a]	1.241	1	.000	-20.49	-12.14
	14.02	-10.53[a]	1.081	1	.000	-14.16	-6.89
	14.03	-7.82[a]	1.277	1	.000	-11.92	-3.72
	14.04	-1.16	.962	1	1.000	-3.60	1.27
	14.05	-5.00[a]	1.000	1	.000	-8.16	-1.83
	14.06	-5.15[a]	1.164	1	.000	-8.78	-1.53
13.08	13.07	-2.94[a]	.734	1	.001	-5.20	-.68
	13.09	-.91	.792	1	1.000	-2.88	1.07
	13.10	-5.17[a]	.789	1	.000	-7.72	-2.62
	13.11	-10.45[a]	.920	1	.000	-13.54	-7.36
	13.12	-14.96[a]	.923	1	.000	-18.06	-11.87
	14.01	-19.26[a]	1.193	1	.000	-23.25	-15.27
	14.02	-13.47[a]	1.025	1	.000	-16.90	-10.04
	14.03	-10.76[a]	1.230	1	.000	-14.87	-6.66
	14.04	-4.11[a]	.899	1	.000	-6.93	-1.29
	14.05	-7.94[a]	.940	1	.000	-11.07	-4.81
	14.06	-8.10[a]	1.113	1	.000	-11.71	-4.49
13.09	13.07	-2.04	.863	1	.218	-4.51	.43
	13.08	.91	.792	1	1.000	-1.07	2.88
	13.10	-4.26[a]	.910	1	.000	-7.12	-1.40
	13.11	-9.54[a]	1.026	1	.000	-12.96	-6.13
	13.12	-14.06[a]	1.028	1	.000	-17.47	-10.64
	14.01	-18.36[a]	1.276	1	.000	-22.59	-14.12
	14.02	-12.56[a]	1.121	1	.000	-16.28	-8.85
	14.03	-9.86[a]	1.311	1	.000	-14.14	-5.57
	14.04	-3.20[a]	1.007	1	.029	-6.24	-.16
	14.05	-7.03[a]	1.043	1	.000	-10.41	-3.65
	14.06	-7.19[a]	1.201	1	.000	-11.04	-3.34
13.10	13.07	2.23	.860	1	.144	-.30	4.75
	13.08	5.17[a]	.789	1	.000	2.62	7.72
	13.09	4.26[a]	.910	1	.000	1.40	7.12
	13.11	-5.28[a]	1.023	1	.000	-8.53	-2.03
	13.12	-9.79[a]	1.026	1	.000	-13.19	-6.40
	14.01	-14.09[a]	1.274	1	.000	-18.30	-9.88
	14.02	-8.30[a]	1.119	1	.000	-11.94	-4.66
	14.03	-5.59[a]	1.309	1	.001	-9.65	-1.53
	14.04	1.06	1.004	1	1.000	-1.39	3.52

	14.05	-2.77	1.041	1	.125	-5.85	.31
	14.06	-2.93	1.199	1	.190	-6.39	.54
13.11	13.07	7.51[a]	.982	1	.000	4.29	10.72
	13.08	10.45[a]	.920	1	.000	7.36	13.54
	13.09	9.54[a]	1.026	1	.000	6.13	12.96
	13.10	5.28[a]	1.023	1	.000	2.03	8.53
	13.12	-4.51[a]	1.130	1	.002	-7.98	-1.05
	14.01	-8.81[a]	1.359	1	.000	-13.20	-4.42
	14.02	-3.02	1.215	1	.181	-6.56	.52
	14.03	-.31	1.392	1	1.000	-3.15	2.53
	14.04	6.34[a]	1.110	1	.000	2.79	9.89
	14.05	2.51	1.144	1	.310	-.74	5.76
	14.06	2.35	1.289	1	.578	-1.20	5.90
13.12	13.07	12.02[a]	.985	1	.000	8.70	15.34
	13.08	14.96[a]	.923	1	.000	11.87	18.06
	13.09	14.06[a]	1.028	1	.000	10.64	17.47
	13.10	9.79[a]	1.026	1	.000	6.40	13.19
	13.11	4.51[a]	1.130	1	.002	1.05	7.98
	14.01	-4.30[a]	1.361	1	.030	-8.39	-.20
	14.02	1.49	1.217	1	1.000	-1.72	4.70
	14.03	4.20[a]	1.394	1	.044	.05	8.35
	14.04	10.86[a]	1.113	1	.000	7.19	14.52
	14.05	7.02[a]	1.146	1	.000	3.33	10.71
	14.06	6.86[a]	1.292	1	.000	2.76	10.97
14.01	13.07	16.32[a]	1.241	1	.000	12.14	20.49
	13.08	19.26[a]	1.193	1	.000	15.27	23.25
	13.09	18.36[a]	1.276	1	.000	14.12	22.59
	13.10	14.09[a]	1.274	1	.000	9.88	18.30
	13.11	8.81[a]	1.359	1	.000	4.42	13.20
	13.12	4.30[a]	1.361	1	.030	.20	8.39
	14.02	5.79[a]	1.433	1	.001	1.36	10.22
	14.03	8.50[a]	1.585	1	.000	3.44	13.55
	14.04	15.15[a]	1.345	1	.000	10.73	19.58
	14.05	11.32[a]	1.373	1	.000	6.81	15.83
	14.06	11.16[a]	1.496	1	.000	6.28	16.04
14.02	13.07	10.53[a]	1.081	1	.000	6.89	14.16
	13.08	13.47[a]	1.025	1	.000	10.04	16.90
	13.09	12.56[a]	1.121	1	.000	8.85	16.28
	13.10	8.30[a]	1.119	1	.000	4.66	11.94
	13.11	3.02	1.215	1	.181	-.52	6.56
	13.12	-1.49	1.217	1	1.000	-4.70	1.72
	14.01	-5.79[a]	1.433	1	.001	-10.22	-1.36
	14.03	2.71	1.464	1	.578	-1.35	6.77
	14.04	9.36[a]	1.199	1	.000	5.43	13.29
	14.05	5.53[a]	1.230	1	.000	1.69	9.37
	14.06	5.37[a]	1.366	1	.002	1.20	9.54
14.03	13.07	7.82[a]	1.277	1	.000	3.72	11.92

	13.08	10.76[a]	1.230	1	.000	6.66	14.87
	13.09	9.86[a]	1.311	1	.000	5.57	14.14
	13.10	5.59[a]	1.309	1	.001	1.53	9.65
	13.11	.31	1.392	1	1.000	-2.53	3.15
	13.12	-4.20[a]	1.394	1	.044	-8.35	-.05
	14.01	-8.50[a]	1.585	1	.000	-13.55	-3.44
	14.02	-2.71	1.464	1	.578	-6.77	1.35
	14.04	6.65[a]	1.378	1	.000	2.31	11.00
	14.05	2.82	1.405	1	.446	-1.12	6.77
	14.06	2.66	1.526	1	.578	-1.45	6.78
14.04	13.07	1.16	.962	1	1.000	-1.27	3.60
	13.08	4.11[a]	.899	1	.000	1.29	6.93
	13.09	3.20[a]	1.007	1	.029	.16	6.24
	13.10	-1.06	1.004	1	1.000	-3.52	1.39
	13.11	-6.34[a]	1.110	1	.000	-9.89	-2.79
	13.12	-10.86[a]	1.113	1	.000	-14.52	-7.19
	14.01	-15.15[a]	1.345	1	.000	-19.58	-10.73
	14.02	-9.36[a]	1.199	1	.000	-13.29	-5.43
	14.03	-6.65[a]	1.378	1	.000	-11.00	-2.31
	14.05	-3.83[a]	1.127	1	.014	-7.26	-.41
	14.06	-3.99[a]	1.274	1	.031	-7.80	-.18
14.05	13.07	5.00[a]	1.000	1	.000	1.83	8.16
	13.08	7.94[a]	.940	1	.000	4.81	11.07
	13.09	7.03[a]	1.043	1	.000	3.65	10.41
	13.10	2.77	1.041	1	.125	-.31	5.85
	13.11	-2.51	1.144	1	.310	-5.76	.74
	13.12	-7.02[a]	1.146	1	.000	-10.71	-3.33
	14.01	-11.32[a]	1.373	1	.000	-15.83	-6.81
	14.02	-5.53[a]	1.230	1	.000	-9.37	-1.69
	14.03	-2.82	1.405	1	.446	-6.77	1.12
	14.04	3.83[a]	1.127	1	.014	.41	7.26
	14.06	-.16	1.303	1	1.000	-2.77	2.45
14.06	13.07	5.15[a]	1.164	1	.000	1.53	8.78
	13.08	8.10[a]	1.113	1	.000	4.49	11.71
	13.09	7.19[a]	1.201	1	.000	3.34	11.04
	13.10	2.93	1.199	1	.190	-.54	6.39
	13.11	-2.35	1.289	1	.578	-5.90	1.20
	13.12	-6.86[a]	1.292	1	.000	-10.97	-2.76
	14.01	-11.16[a]	1.496	1	.000	-16.04	-6.28
	14.02	-5.37[a]	1.366	1	.002	-9.54	-1.20
	14.03	-2.66	1.526	1	.578	-6.78	1.45
	14.04	3.99[a]	1.274	1	.031	.18	7.80
	14.05	.16	1.303	1	1.000	-2.45	2.77

Pairwise comparisons of estimated marginal means based on the original scale of dependent variable Duration

a. The mean difference is significant at the .05 level.

b. Confidence interval bounds are approximate.

- **Dependent Variable: Swarm size; Predictor: Month**

Model Information

Dependent Variable	Size
Probability Distribution	Negative binomial (1)
Link Function	Log

Continuous Variable Information

		N	Minimum	Maximum	Mean	Std. Deviation
Dependent Variable	Size	480	4	675	118.55	132.522

Goodness of Fit[a]

	Value	df	Value/df
Deviance	382.304	468	.817
Scaled Deviance	382.304	468	
Pearson Chi-Square	398.146	468	.851
Scaled Pearson Chi-Square	398.146	468	
Log Likelihood[b]	-2679.432		
Akaike's Information Criterion (AIC)	5382.864		
Finite Sample Corrected AIC (AICC)	5383.533		
Bayesian Information Criterion (BIC)	5432.950		
Consistent AIC (CAIC)	5444.950		

Model: (Intercept). Month[a]
a. Information criteria are in smaller-is-better form.
b. The full log likelihood function is displayed and used in computing information criteria.

Omnibus Test[a]

Likelihood Ratio Chi-Square	df	Sig.
189.511	11	.000

Model: (Intercept). Month[a]
a. Compares the fitted model against the intercept-only model.

Tests of Model Effects

Source	Type III

	Wald Chi-Square	df	Sig.
(Intercept)	11473.269	1	.000
Month	401.977	11	.000

Model: (Intercept). Month

Estimated Marginal Means: Month

Month	Mean	Std. Error	95% Wald Confidence Interval	
			Lower	Upper
13.07	72.30	8.017	58.18	89.86
13.08	19.78	2.512	15.42	25.37
13.09	36.50	5.394	27.32	48.76
13.10	157.87	18.793	125.01	199.35
13.11	260.91	30.916	206.84	329.12
13.12	155.18	16.980	125.22	192.29
14.01	104.74	15.566	78.27	140.15
14.02	97.46	13.217	74.71	127.13
14.03	225.18	23.787	183.07	276.98
14.04	42.29	6.829	30.82	58.04
14.05	98.73	21.385	64.58	150.95
14.06	93.77	18.671	63.47	138.53

Pairwise Comparisons

(I) Month	(J) Month	Mean Difference (I-J)	Std. Error	df	Sequential Bonferroni Sig.	95% Wald Confidence Interval for Difference[b]	
						Lower	Upper
13.07	13.08	52.52[a]	8.402	1	.000	24.57	80.48
	13.09	35.80[a]	9.663	1	.008	4.94	66.66
	13.10	-85.56[a]	20.432	1	.001	-151.50	-19.63
	13.11	-188.61[a]	31.939	1	.000	-294.23	-82.99
	13.12	-82.87[a]	18.777	1	.000	-144.10	-21.65
	14.01	-32.43	17.509	1	.832	-83.04	18.18
	14.02	-25.15	15.459	1	1.000	-69.45	19.14
	14.03	-152.88[a]	25.102	1	.000	-236.02	-69.74
	14.04	30.01	10.531	1	.127	-3.00	63.02
	14.05	-26.43	22.839	1	1.000	-83.56	30.71
	14.06	-21.47	20.320	1	1.000	-71.12	28.19
13.08	13.07	-52.52[a]	8.402	1	.000	-80.48	-24.57
	13.09	-16.72	5.950	1	.129	-35.18	1.74
	13.10	-138.09[a]	18.961	1	.000	-201.53	-74.64
	13.11	-241.13[a]	31.018	1	.000	-345.33	-136.93
	13.12	-135.40[a]	17.164	1	.000	-193.13	-77.66
	14.01	-84.96[a]	15.767	1	.000	-136.84	-33.07
	14.02	-77.68[a]	13.454	1	.000	-122.02	-33.33

	14.03	-205.40[a]	23.919	1	.000	-285.96	-124.84
	14.04	-22.51	7.276	1	.063	-45.52	.50
	14.05	-78.95[a]	21.532	1	.008	-147.25	-10.64
	14.06	-73.99[a]	18.840	1	.003	-134.37	-13.61
13.09	13.07	-35.80[a]	9.663	1	.008	-66.66	-4.94
	13.08	16.72	5.950	1	.129	-1.74	35.18
	13.10	-121.37[a]	19.552	1	.000	-186.33	-56.41
	13.11	-224.41[a]	31.383	1	.000	-329.28	-119.55
	13.12	-118.68[a]	17.816	1	.000	-178.04	-59.31
	14.01	-68.24[a]	16.474	1	.001	-121.28	-15.19
	14.02	-60.96[a]	14.276	1	.001	-107.23	-14.69
	14.03	-188.68[a]	24.391	1	.000	-270.51	-106.85
	14.04	-5.79	8.702	1	1.000	-25.26	13.67
	14.05	-62.23	22.055	1	.129	-130.89	6.43
	14.06	-57.27	19.435	1	.096	-118.37	3.83
13.10	13.07	85.56[a]	20.432	1	.001	19.63	151.50
	13.08	138.09[a]	18.961	1	.000	74.64	201.53
	13.09	121.37[a]	19.552	1	.000	56.41	186.33
	13.11	-103.04	36.180	1	.127	-216.38	10.29
	13.12	2.69	25.328	1	1.000	-47.90	53.28
	14.01	53.13	24.402	1	.485	-19.20	125.46
	14.02	60.41	22.976	1	.205	-10.31	131.13
	14.03	-67.31	30.315	1	.475	-158.00	23.37
	14.04	115.57[a]	19.996	1	.000	49.56	181.59
	14.05	59.14	28.470	1	.567	-24.43	142.70
	14.06	64.10	26.492	1	.314	-16.09	144.28
13.11	13.07	188.61[a]	31.939	1	.000	82.99	294.23
	13.08	241.13[a]	31.018	1	.000	136.93	345.33
	13.09	224.41[a]	31.383	1	.000	119.55	329.28
	13.10	103.04	36.180	1	.127	-10.29	216.38
	13.12	105.73	35.272	1	.084	-5.50	216.97
	14.01	156.17[a]	34.613	1	.000	43.09	269.26
	14.02	163.45[a]	33.623	1	.000	53.01	273.90
	14.03	35.73	39.008	1	1.000	-56.56	128.02
	14.04	218.62[a]	31.661	1	.000	112.97	324.27
	14.05	162.18[a]	37.592	1	.001	40.09	284.27
	14.06	167.14[a]	36.117	1	.000	48.93	285.35
13.12	13.07	82.87[a]	18.777	1	.000	21.65	144.10
	13.08	135.40[a]	17.164	1	.000	77.66	193.13
	13.09	118.68[a]	17.816	1	.000	59.31	178.04
	13.10	-2.69	25.328	1	1.000	-53.28	47.90
	13.11	-105.73	35.272	1	.084	-216.97	5.50
	14.01	50.44	23.035	1	.485	-18.06	118.94
	14.02	57.72	21.518	1	.183	-8.77	124.21
	14.03	-70.00	29.226	1	.316	-157.91	17.90
	14.04	112.88[a]	18.301	1	.000	52.18	173.59
	14.05	56.45	27.306	1	.567	-23.49	136.39

	14.06	61.41	25.237	1	.314	-15.27	138.08
14.01	13.07	32.43	17.509	1	.832	-18.18	83.04
	13.08	84.96[a]	15.767	1	.000	33.07	136.84
	13.09	68.24[a]	16.474	1	.001	15.19	121.28
	13.10	-53.13	24.402	1	.485	-125.46	19.20
	13.11	-156.17[a]	34.613	1	.000	-269.26	-43.09
	13.12	-50.44	23.035	1	.485	-118.94	18.06
	14.02	7.28	20.420	1	1.000	-35.52	50.08
	14.03	-120.44[a]	28.427	1	.001	-212.39	-28.50
	14.04	62.44[a]	16.998	1	.008	8.38	116.50
	14.05	6.01	26.450	1	1.000	-48.04	60.05
	14.06	10.97	24.309	1	1.000	-40.98	62.91
14.02	13.07	25.15	15.459	1	1.000	-19.14	69.45
	13.08	77.68[a]	13.454	1	.000	33.33	122.02
	13.09	60.96[a]	14.276	1	.001	14.69	107.23
	13.10	-60.41	22.976	1	.205	-131.13	10.31
	13.11	-163.45[a]	33.623	1	.000	-273.90	-53.01
	13.12	-57.72	21.518	1	.183	-124.21	8.77
	14.01	-7.28	20.420	1	1.000	-50.08	35.52
	14.03	-127.72[a]	27.213	1	.000	-216.96	-38.49
	14.04	55.16[a]	14.877	1	.008	7.60	102.73
	14.05	-1.27	25.140	1	1.000	-50.99	48.44
	14.06	3.69	22.876	1	1.000	-42.48	49.85
14.03	13.07	152.88[a]	25.102	1	.000	69.74	236.02
	13.08	205.40[a]	23.919	1	.000	124.84	285.96
	13.09	188.68[a]	24.391	1	.000	106.85	270.51
	13.10	67.31	30.315	1	.475	-23.37	158.00
	13.11	-35.73	39.008	1	1.000	-128.02	56.56
	13.12	70.00	29.226	1	.316	-17.90	157.91
	14.01	120.44[a]	28.427	1	.001	28.50	212.39
	14.02	127.72[a]	27.213	1	.000	38.49	216.96
	14.04	182.89[a]	24.748	1	.000	99.97	265.81
	14.05	126.45[a]	31.987	1	.003	23.69	229.21
	14.06	131.41[a]	30.240	1	.001	33.00	229.82
14.04	13.07	-30.01	10.531	1	.127	-63.02	3.00
	13.08	22.51	7.276	1	.063	-.50	45.52
	13.09	5.79	8.702	1	1.000	-13.67	25.26
	13.10	-115.57[a]	19.996	1	.000	-181.59	-49.56
	13.11	-218.62[a]	31.661	1	.000	-324.27	-112.97
	13.12	-112.88[a]	18.301	1	.000	-173.59	-52.18
	14.01	-62.44[a]	16.998	1	.008	-116.50	-8.38
	14.02	-55.16[a]	14.877	1	.008	-102.73	-7.60
	14.03	-182.89[a]	24.748	1	.000	-265.81	-99.97
	14.05	-56.44	22.449	1	.263	-124.95	12.08
	14.06	-51.48	19.881	1	.221	-112.42	9.46
14.05	13.07	26.43	22.839	1	1.000	-30.71	83.56
	13.08	78.95[a]	21.532	1	.008	10.64	147.25

	13.09	62.23	22.055	1	.129	-6.43	130.89
	13.10	-59.14	28.470	1	.567	-142.70	24.43
	13.11	-162.18[a]	37.592	1	.001	-284.27	-40.09
	13.12	-56.45	27.306	1	.567	-136.39	23.49
	14.01	-6.01	26.450	1	1.000	-60.05	48.04
	14.02	1.27	25.140	1	1.000	-48.44	50.99
	14.03	-126.45[a]	31.987	1	.003	-229.21	-23.69
	14.04	56.44	22.449	1	.263	-12.08	124.95
	14.06	4.96	28.389	1	1.000	-52.47	62.39
14.06	13.07	21.47	20.320	1	1.000	-28.19	71.12
	13.08	73.99[a]	18.840	1	.003	13.61	134.37
	13.09	57.27	19.435	1	.096	-3.83	118.37
	13.10	-64.10	26.492	1	.314	-144.28	16.09
	13.11	-167.14[a]	36.117	1	.000	-285.35	-48.93
	13.12	-61.41	25.237	1	.314	-138.08	15.27
	14.01	-10.97	24.309	1	1.000	-62.91	40.98
	14.02	-3.69	22.876	1	1.000	-49.85	42.48
	14.03	-131.41[a]	30.240	1	.001	-229.82	-33.00
	14.04	51.48	19.881	1	.221	-9.46	112.42
	14.05	-4.96	28.389	1	1.000	-62.39	52.47

Pairwise comparisons of estimated marginal means based on the original scale of dependent variable Size

a. The mean difference is significant at the .05 level.

b. Confidence interval bounds are approximate.

- **Dependent Variable: Number of mating pairs per swarm; Predictor: Month**

Model Information

Dependent Variable	Mating pairs
Probability Distribution	Negative binomial (1)
Link Function	Log

Continuous Variable Information

		N	Minimum	Maximum	Mean	Std. Deviation
Dependent Variable	Mating pairs	458	0	287	23.03	39.159

Goodness of Fit[a]

	Value	df	Value/df
Deviance	786.926	446	1.764
Scaled Deviance	786.926	446	
Pearson Chi-Square	941.716	446	2.111
Scaled Pearson Chi-Square	941.716	446	
Log Likelihood[b]	-1608.196		

Akaike's Information Criterion (AIC)	3240.392		
Finite Sample Corrected AIC (AICC)	3241.094		
Bayesian Information Criterion (BIC)	3289.915		
Consistent AIC (CAIC)	3301.915		

Model: (Intercept). Month[a]
a. Information criteria are in smaller-is-better form.
b. The full log likelihood function is displayed and used in computing information criteria.

Omnibus Test[a]

Likelihood Ratio Chi-Square	df	Sig.
592.712	11	.000

Model: (Intercept). Month[a]
a. Compares the fitted model against the intercept-only model.

Tests of Model Effects

Source	Type III		
	Wald Chi-Square	df	Sig.
(Intercept)	1250.908	1	.000
Month	95.123	9	.000

Model: (Intercept). Month

Estimated Marginal Means: Month

Month	Mean	Std. Error	95% Wald Confidence Interval	
			Lower	Upper
13.07	4.82	1.902	2.22	10.45
13.08	.00	.000	.00	.00
13.09	.00	.000	.00	.00
13.10	19.38	3.766	13.24	28.37
13.11	45.88	5.892	35.67	59.01
13.12	27.31	5.181	18.83	39.61
14.01	23.53	5.843	14.46	38.28
14.02	30.23	5.626	20.99	43.54
14.03	79.97	12.319	59.13	108.16
14.04	8.03	3.324	3.57	18.07
14.05	20.08	4.956	12.38	32.57
14.06	13.76	5.083	6.67	28.38

Pairwise Comparisons

(I) Month	(J) Month	Mean Difference (I-J)	Std. Error	df	Sequential Bonferroni Sig.	95% Wald Confidence Interval for Difference[a]	
						Lower	Upper
13.07	13.08	4.82	1.902	1	.328	-1.14	10.78
	13.09	4.82	1.902	1	.328	-1.14	10.78
	13.10	-14.57[b]	4.219	1	.021	-28.12	-1.01
	13.11	-41.06[b]	6.192	1	.000	-61.87	-20.26
	13.12	-22.49[b]	5.519	1	.002	-40.52	-4.46
	14.01	-18.71	6.145	1	.079	-38.25	.84
	14.02	-25.41[b]	5.939	1	.001	-44.92	-5.90
	14.03	-75.15[b]	12.465	1	.000	-116.86	-33.44
	14.04	-3.21	3.830	1	1.000	-12.11	5.69
	14.05	-15.26	5.308	1	.133	-32.10	1.57
	14.06	-8.94	5.428	1	1.000	-24.19	6.30
13.08	13.07	-4.82	1.902	1	.328	-10.78	1.14
	13.09	.00	.000	1	.	.00	.00
	13.10	-19.38[b]	3.766	1	.000	-31.86	-6.91
	13.11	-45.88[b]	5.892	1	.000	-65.73	-26.04
	13.12	-27.31[b]	5.181	1	.000	-44.52	-10.09
	14.01	-23.53[b]	5.843	1	.002	-42.51	-4.55
	14.02	-30.23[b]	5.626	1	.000	-48.98	-11.48
	14.03	-79.97[b]	12.319	1	.000	-121.30	-38.64
	14.04	-8.03	3.324	1	.409	-18.34	2.28
	14.05	-20.08[b]	4.956	1	.002	-36.24	-3.92
	14.06	-13.76	5.083	1	.217	-29.84	2.32
13.09	13.07	-4.82	1.902	1	.328	-10.78	1.14
	13.08	.00	.000	1	.	.00	.00
	13.10	-19.38[b]	3.766	1	.000	-31.86	-6.91
	13.11	-45.88[b]	5.892	1	.000	-65.73	-26.04
	13.12	-27.31[b]	5.181	1	.000	-44.52	-10.09
	14.01	-23.53[b]	5.843	1	.002	-42.51	-4.55
	14.02	-30.23[b]	5.626	1	.000	-48.98	-11.48
	14.03	-79.97[b]	12.319	1	.000	-121.30	-38.64
	14.04	-8.03	3.324	1	.409	-18.34	2.28
	14.05	-20.08[b]	4.956	1	.002	-36.24	-3.92
	14.06	-13.76	5.083	1	.217	-29.84	2.32
13.10	13.07	14.57[b]	4.219	1	.021	1.01	28.12
	13.08	19.38[b]	3.766	1	.000	6.91	31.86
	13.09	19.38[b]	3.766	1	.000	6.91	31.86
	13.11	-26.50[b]	6.993	1	.006	-49.07	-3.93
	13.12	-7.92	6.405	1	1.000	-24.25	8.40
	14.01	-4.14	6.952	1	1.000	-19.46	11.18
	14.02	-10.85	6.771	1	1.000	-29.66	7.97
	14.03	-60.59[b]	12.882	1	.000	-103.05	-18.13
	14.04	11.36	5.023	1	.523	-3.97	26.69
	14.05	-.70	6.224	1	1.000	-13.14	11.75

	14.06	5.62	6.326	1	1.000	-9.25	20.50
13.11	13.07	41.06[b]	6.192	1	.000	20.26	61.87
	13.08	45.88[b]	5.892	1	.000	26.04	65.73
	13.09	45.88[b]	5.892	1	.000	26.04	65.73
	13.10	26.50[b]	6.993	1	.006	3.93	49.07
	13.12	18.58	7.846	1	.430	-5.58	42.73
	14.01	22.36	8.299	1	.217	-3.80	48.51
	14.02	15.65	8.147	1	1.000	-8.85	40.16
	14.03	-34.09	13.656	1	.339	-76.60	8.42
	14.04	37.86[b]	6.765	1	.000	15.28	60.43
	14.05	25.80[b]	7.699	1	.029	1.19	50.42
	14.06	32.12[b]	7.782	1	.002	6.65	57.59
13.12	13.07	22.49[b]	5.519	1	.002	4.46	40.52
	13.08	27.31[b]	5.181	1	.000	10.09	44.52
	13.09	27.31[b]	5.181	1	.000	10.09	44.52
	13.10	7.92	6.405	1	1.000	-8.40	24.25
	13.11	-18.58	7.846	1	.430	-42.73	5.58
	14.01	3.78	7.810	1	1.000	-13.02	20.58
	14.02	-2.92	7.649	1	1.000	-19.04	13.19
	14.03	-52.66[b]	13.364	1	.003	-95.89	-9.44
	14.04	19.28	6.156	1	.061	-.35	38.91
	14.05	7.23	7.170	1	1.000	-10.10	24.55
	14.06	13.55	7.259	1	1.000	-7.92	35.01
14.01	13.07	18.71	6.145	1	.079	-.84	38.25
	13.08	23.53[b]	5.843	1	.002	4.55	42.51
	13.09	23.53[b]	5.843	1	.002	4.55	42.51
	13.10	4.14	6.952	1	1.000	-11.18	19.46
	13.11	-22.36	8.299	1	.217	-48.51	3.80
	13.12	-3.78	7.810	1	1.000	-20.58	13.02
	14.02	-6.70	8.112	1	1.000	-25.51	12.10
	14.03	-56.44[b]	13.635	1	.002	-101.15	-11.73
	14.04	15.50	6.723	1	.486	-5.11	36.11
	14.05	3.45	7.662	1	1.000	-12.92	19.81
	14.06	9.77	7.745	1	1.000	-10.09	29.62
14.02	13.07	25.41[b]	5.939	1	.001	5.90	44.92
	13.08	30.23[b]	5.626	1	.000	11.48	48.98
	13.09	30.23[b]	5.626	1	.000	11.48	48.98
	13.10	10.85	6.771	1	1.000	-7.97	29.66
	13.11	-15.65	8.147	1	1.000	-40.16	8.85
	13.12	2.92	7.649	1	1.000	-13.19	19.04
	14.01	6.70	8.112	1	1.000	-12.10	25.51
	14.03	-49.74[b]	13.543	1	.009	-93.35	-6.13
	14.04	22.20[b]	6.535	1	.025	1.26	43.15
	14.05	10.15	7.498	1	1.000	-9.50	29.79
	14.06	16.47	7.583	1	.627	-6.57	39.51
14.03	13.07	75.15[b]	12.465	1	.000	33.44	116.86
	13.08	79.97[b]	12.319	1	.000	38.64	121.30

	13.09	79.97[b]	12.319	1	.000	38.64	121.30
	13.10	60.59[b]	12.882	1	.000	18.13	103.05
	13.11	34.09	13.656	1	.339	-8.42	76.60
	13.12	52.66[b]	13.364	1	.003	9.44	95.89
	14.01	56.44[b]	13.635	1	.002	11.73	101.15
	14.02	49.74[b]	13.543	1	.009	6.13	93.35
	14.04	71.94[b]	12.760	1	.000	29.31	114.58
	14.05	59.89[b]	13.279	1	.000	16.20	103.58
	14.06	66.21[b]	13.327	1	.000	22.21	110.21
14.04	13.07	3.21	3.830	1	1.000	-5.69	12.11
	13.08	8.03	3.324	1	.409	-2.28	18.34
	13.09	8.03	3.324	1	.409	-2.28	18.34
	13.10	-11.36	5.023	1	.523	-26.69	3.97
	13.11	-37.86[b]	6.765	1	.000	-60.43	-15.28
	13.12	-19.28	6.156	1	.061	-38.91	.35
	14.01	-15.50	6.723	1	.486	-36.11	5.11
	14.02	-22.20[b]	6.535	1	.025	-43.15	-1.26
	14.03	-71.94[b]	12.760	1	.000	-114.58	-29.31
	14.05	-12.05	5.967	1	.868	-30.09	5.99
	14.06	-5.73	6.074	1	1.000	-20.19	8.73
14.05	13.07	15.26	5.308	1	.133	-1.57	32.10
	13.08	20.08[b]	4.956	1	.002	3.92	36.24
	13.09	20.08[b]	4.956	1	.002	3.92	36.24
	13.10	.70	6.224	1	1.000	-11.75	13.14
	13.11	-25.80[b]	7.699	1	.029	-50.42	-1.19
	13.12	-7.23	7.170	1	1.000	-24.55	10.10
	14.01	-3.45	7.662	1	1.000	-19.81	12.92
	14.02	-10.15	7.498	1	1.000	-29.79	9.50
	14.03	-59.89[b]	13.279	1	.000	-103.58	-16.20
	14.04	12.05	5.967	1	.868	-5.99	30.09
	14.06	6.32	7.099	1	1.000	-10.38	23.02
14.06	13.07	8.94	5.428	1	1.000	-6.30	24.19
	13.08	13.76	5.083	1	.217	-2.32	29.84
	13.09	13.76	5.083	1	.217	-2.32	29.84
	13.10	-5.62	6.326	1	1.000	-20.50	9.25
	13.11	-32.12[b]	7.782	1	.002	-57.59	-6.65
	13.12	-13.55	7.259	1	1.000	-35.01	7.92
	14.01	-9.77	7.745	1	1.000	-29.62	10.09
	14.02	-16.47	7.583	1	.627	-39.51	6.57
	14.03	-66.21[b]	13.327	1	.000	-110.21	-22.21
	14.04	5.73	6.074	1	1.000	-8.73	20.19
	14.05	-6.32	7.099	1	1.000	-23.02	10.38

Pairwise comparisons of estimated marginal means based on the original scale of dependent variable Mating pairs

a. Confidence interval bounds are approximate.

b. The mean difference is significant at the .05 level.

- **Dependent Variable: Time to first mating pair; Predictor: Month**

Model Information

Dependent Variable	Time to first mating
Probability Distribution	Gamma
Link Function	Log

Continuous Variable Information

		N	Minimum	Maximum	Mean	Std. Deviation
Dependent Variable	Time to first mating	310	2	34	13.32	6.425

Goodness of Fit[a]

	Value	df	Value/df
Deviance	63.908	300	.213
Scaled Deviance	320.270	300	
Pearson Chi-Square	51.811	300	.173
Scaled Pearson Chi-Square	259.646	300	
Log Likelihood[b]	-957.709		
Akaike's Information Criterion (AIC)	1937.418		
Finite Sample Corrected AIC (AICC)	1938.304		
Bayesian Information Criterion (BIC)	1978.520		
Consistent AIC (CAIC)	1989.520		

Model: (Intercept), Month[a]
a. Information criteria are in smaller-is-better form.
b. The full log likelihood function is displayed and used in computing information criteria.

Omnibus Test[a]

Likelihood Ratio Chi-Square	df	Sig.
112.891	9	.000

Model: (Intercept), Month[a]
a. Compares the fitted model against the intercept-only model.

Tests of Model Effects

	Type III		
Source	Wald Chi-Square	df	Sig.

(Intercept)	10944.779	1	.000
Month	237.597	9	.000

Model: (Intercept), Month

Estimated Marginal Means: Month

Month	Mean	Std. Error	95% Wald Confidence Interval	
			Lower	Upper
13.07	11.23	1.094	9.28	13.59
13.10	7.98	.665	6.77	9.39
13.11	10.38	.853	8.84	12.20
13.12	16.34	.805	14.84	18.00
14.01	21.49	.762	20.04	23.03
14.02	14.61	.955	12.85	16.60
14.03	10.84	.965	9.10	12.90
14.04	13.00	.862	11.42	14.80
14.05	11.23	.687	9.96	12.66
14.06	11.75	1.224	9.58	14.41

Pairwise Comparisons

(I) Month	(J) Month	Mean Difference (I-J)	Std. Error	df	Sequential Bonferroni Sig.	95% Wald Confidence Interval for Difference[a]	
						Lower	Upper
13.07	13.10	3.25	1.280	1	.242	-.65	7.16
	13.11	.85	1.388	1	1.000	-2.22	3.92
	13.12	-5.11[b]	1.358	1	.005	-9.39	-.84
	14.01	-10.26[b]	1.333	1	.000	-14.56	-5.95
	14.02	-3.38	1.452	1	.403	-7.77	1.02
	14.03	.39	1.459	1	1.000	-2.61	3.40
	14.04	-1.77	1.393	1	1.000	-5.35	1.81
	14.05	.00	1.292	1	1.000	-2.53	2.53
	14.06	-.52	1.642	1	1.000	-3.93	2.90
13.10	13.07	-3.25	1.280	1	.242	-7.16	.65
	13.11	-2.41	1.082	1	.496	-5.66	.85
	13.12	-8.37[b]	1.044	1	.000	-11.75	-4.99
	14.01	-13.51[b]	1.011	1	.000	-16.81	-10.21
	14.02	-6.63[b]	1.164	1	.000	-10.36	-2.90
	14.03	-2.86	1.171	1	.305	-6.42	.70
	14.04	-5.02[b]	1.088	1	.000	-8.47	-1.58
	14.05	-3.25[b]	.955	1	.019	-6.25	-.26
	14.06	-3.77	1.393	1	.155	-8.04	.50
13.11	13.07	-.85	1.388	1	1.000	-3.92	2.22
	13.10	2.41	1.082	1	.496	-.85	5.66
	13.12	-5.96[b]	1.173	1	.000	-9.70	-2.22

	14.01	-11.10[b]	1.144	1	.000	-14.83	-7.38
	14.02	-4.22[b]	1.281	1	.027	-8.23	-.22
	14.03	-.46	1.288	1	1.000	-3.15	2.24
	14.04	-2.62	1.213	1	.556	-6.25	1.01
	14.05	-.85	1.095	1	1.000	-3.36	1.66
	14.06	-1.37	1.493	1	1.000	-4.90	2.16
13.12	13.07	5.11[b]	1.358	1	.005	.84	9.39
	13.10	8.37[b]	1.044	1	.000	4.99	11.75
	13.11	5.96[b]	1.173	1	.000	2.22	9.70
	14.01	-5.14[b]	1.109	1	.000	-8.66	-1.63
	14.02	1.74	1.249	1	1.000	-1.56	5.04
	14.03	5.51[b]	1.257	1	.000	1.54	9.47
	14.04	3.34	1.179	1	.114	-.30	6.99
	14.05	5.11[b]	1.058	1	.000	1.75	8.48
	14.06	4.59[b]	1.466	1	.046	.03	9.16
14.01	13.07	10.26[b]	1.333	1	.000	5.95	14.56
	13.10	13.51[b]	1.011	1	.000	10.21	16.81
	13.11	11.10[b]	1.144	1	.000	7.38	14.83
	13.12	5.14[b]	1.109	1	.000	1.63	8.66
	14.02	6.88[b]	1.222	1	.000	2.97	10.79
	14.03	10.65[b]	1.229	1	.000	6.65	14.64
	14.04	8.49[b]	1.150	1	.000	4.78	12.19
	14.05	10.26[b]	1.026	1	.000	6.93	13.58
	14.06	9.74[b]	1.442	1	.000	5.10	14.37
14.02	13.07	3.38	1.452	1	.403	-1.02	7.77
	13.10	6.63[b]	1.164	1	.000	2.90	10.36
	13.11	4.22[b]	1.281	1	.027	.22	8.23
	13.12	-1.74	1.249	1	1.000	-5.04	1.56
	14.01	-6.88[b]	1.222	1	.000	-10.79	-2.97
	14.03	3.77	1.358	1	.132	-.41	7.95
	14.04	1.61	1.286	1	1.000	-1.68	4.89
	14.05	3.38	1.176	1	.107	-.27	7.02
	14.06	2.86	1.553	1	1.000	-1.76	7.47
14.03	13.07	-.39	1.459	1	1.000	-3.40	2.61
	13.10	2.86	1.171	1	.305	-.70	6.42
	13.11	.46	1.288	1	1.000	-2.24	3.15
	13.12	-5.51[b]	1.257	1	.000	-9.47	-1.54
	14.01	-10.65[b]	1.229	1	.000	-14.64	-6.65
	14.02	-3.77	1.358	1	.132	-7.95	.41
	14.04	-2.16	1.293	1	1.000	-5.81	1.49
	14.05	-.39	1.184	1	1.000	-2.86	2.08
	14.06	-.91	1.559	1	1.000	-4.34	2.52
14.04	13.07	1.77	1.393	1	1.000	-1.81	5.35
	13.10	5.02[b]	1.088	1	.000	1.58	8.47
	13.11	2.62	1.213	1	.556	-1.01	6.25
	13.12	-3.34	1.179	1	.114	-6.99	.30
	14.01	-8.49[b]	1.150	1	.000	-12.19	-4.78

	14.02	-1.61	1.286	1	1.000	-4.89	1.68
	14.03	2.16	1.293	1	1.000	-1.49	5.81
	14.05	1.77	1.102	1	1.000	-1.29	4.83
	14.06	1.25	1.497	1	1.000	-2.23	4.73
14.05	13.07	.00	1.292	1	1.000	-2.53	2.53
	13.10	3.25[b]	.955	1	.019	.26	6.25
	13.11	.85	1.095	1	1.000	-1.66	3.36
	13.12	-5.11[b]	1.058	1	.000	-8.48	-1.75
	14.01	-10.26[b]	1.026	1	.000	-13.58	-6.93
	14.02	-3.38	1.176	1	.107	-7.02	.27
	14.03	.39	1.184	1	1.000	-2.08	2.86
	14.04	-1.77	1.102	1	1.000	-4.83	1.29
	14.06	-.52	1.404	1	1.000	-3.47	2.43
14.06	13.07	.52	1.642	1	1.000	-2.90	3.93
	13.10	3.77	1.393	1	.155	-.50	8.04
	13.11	1.37	1.493	1	1.000	-2.16	4.90
	13.12	-4.59[b]	1.466	1	.046	-9.16	-.03
	14.01	-9.74[b]	1.442	1	.000	-14.37	-5.10
	14.02	-2.86	1.553	1	1.000	-7.47	1.76
	14.03	.91	1.559	1	1.000	-2.52	4.34
	14.04	-1.25	1.497	1	1.000	-4.73	2.23
	14.05	.52	1.404	1	1.000	-2.43	3.47

Pairwise comparisons of estimated marginal means based on the original scale of dependent variable Time to first mating
a. Confidence interval bounds are approximate.
b. The mean difference is significant at the .05 level.

- **Dependent Variable: Wing length; Predictor: Mosquito group**

Model Information

Dependent Variable	Wing length
Probability Distribution	Gamma
Link Function	Log

Goodness of Fit[a]

	Value	df	Value/df
Deviance	.313	162	.002
Scaled Deviance	165.052	162	
Pearson Chi-Square	.309	162	.002
Scaled Pearson Chi-Square	163.243	162	
Log Likelihood[b]	95.236		
Akaike's Information Criterion (AIC)	-182.472		
Finite Sample Corrected AIC (AICC)	-182.222		

Bayesian Information Criterion (BIC)	-170.049		
Consistent AIC (CAIC)	-166.049		

Model: (Intercept). Mosquitogroup[a]

a. Information criteria are in smaller-is-better form.

b. The full log likelihood function is displayed and used in computing information criteria.

Omnibus Test[a]

Likelihood Ratio Chi-Square	df	Sig.
96.092	2	.000

Model: (Intercept). Mosquito group[a]

a. Compares the fitted model against the intercept-only model.

Tests of Model Effects

Source	Type III		
	Wald Chi-Square	df	Sig.
(Intercept)	114133.979	1	.000
Mosquito group	98.099	2	.000

Model: (Intercept). Mosquito group

Estimated Marginal Means: Mosquito group

Mosquitogroup	Mean	Std. Error	95% Wald Confidence Interval	
			Lower	Upper
Females.in.copula	3.29729	.024764	3.24911	3.34619
Male.in.copula	3.05055	.013562	3.02408	3.07724
Males.in.solo	3.02807	.015467	2.99791	3.05854

\Pairwise Comparisons

(I) Mosquitogroup	(J) Mosquitogroup	Mean Difference (I-J)	Std. Error	df	Sequential Bonferroni Sig.
Females.in.copula	Male.in.copula	.24675[a]	.028234	1	.000
	Males.in.solo	.26922[a]	.029197	1	.000
Male.in.copula	Females.in.copula	-.24675[a]	.028234	1	.000
	Males.in.solo	.02247	.020571	1	.275
Males.in.solo	Females.in.copula	-.26922[a]	.029197	1	.000

Male.in.copula	-.02247	.020571	1	.275

Pairwise Comparisons

(I) Mosquitogroup	(J) Mosquitogroup	95% Wald Confidence Interval for Difference[b] Lower	Upper
Females.in.copula	Male.in.copula	.17915	.31434
	Males.in.solo	.20378	.33466
Male.in.copula	Females.in.copula	-.31434	-.17915
	Males.in.solo	-.01785	.06279
Males.in.solo	Females.in.copula	-.33466	-.20378
	Male.in.copula	-.06279	.01785

Pairwise comparisons of estimated marginal means based on the original scale of dependent variable Winglength

a. The mean difference is significant at the .05 level.

b. Confidence interval bounds are approximate.
